# Test-Retest Reliability of Large Language Models and Their Concordance With Human Experts in Rubric-Based Scoring of Clinical Summary Statements: Experimental Comparative Study

**DOI:** 10.2196/94666

**Published:** 2026-10-21

**Authors:** Patrick Stadler, Piotr Bucichowski, Ylva Holzhausen, Inga Hege, Harm Peters, Julius Josef Kaminski

**Affiliations:** 1 Charité – University Medical Center Berlin, Dieter Scheffner Center for Medical Education and Educational Research, Dean's Office for Study Affairs, Charitéplatz 1, Berlin, 10117, Germany, 49 30450576207; 2 Faculty of Health Sciences Brandenburg, Institute of Research in Health Sciences Education, Brandenburg Medical School, Neuruppin, Germany

**Keywords:** medical education, large language models, automated assessment, formative feedback, clinical reasoning, virtual patients, summary statements, problem representation

## Abstract

**Background:**

Clinical summary statements convert unstructured patient information into a concise format. They are widely used in virtual patients (VPs) to train medical students in diagnostic reasoning. Most VP systems provide little individualized feedback on the quality of learners’ summaries, and earlier automated approaches have required complex natural language processing, limiting their scalability. Large language models (LLMs) may offer a more flexible, rubric-guided way to score summary statements and support feedback. Still, their test-retest reliability and concordance with expert ratings in this context remain unclear.

**Objective:**

This study aims to evaluate the test-retest reliability—specifically, whether the models produce stable ratings across repetitions—of LLM-generated medical summary statement ratings, and to measure their concordance with human expert assessments across different prompting strategies.

**Methods:**

We conducted an experimental comparative study using 122 rubric-scored summary statements (English and German) from 6 internal medicine VP cases previously rated by 2 expert raters. Statements were evaluated along 4 ternary components (semantic qualifiers, transformation, narrowing, and global rating), and 2 binary components (factual accuracy and person). Four LLMs (Anthropic’s Claude, OpenAI’s GPT-4o, Mistral AI’s Mistral Large, and Mistral AI’s Mistral 7B) were each run 5 times under 4 prompting strategies (zero-shot, zero-shot with chain-of-thought [CoT], few-shot, and few-shot+CoT). The test-retest reliability was assessed with intraclass correlation coefficients (ICCs), Fleiss kappa (κ), and concordance with human ratings with accuracy (%) and chance-corrected interrater agreement (Gwet AC1 and AC2).

**Results:**

Large closed-weights models (Claude, GPT-4o, and Mistral Large) showed high test-retest reliability (ICC≥0.92; Fleiss κ=0.59‐1.00). Claude was essentially deterministic (ICC and Fleiss κ≥0.99); GPT-4o and Mistral Large reached ICC≥0.97 and Fleiss κ≥0.80 except under zero-shot. Mistral 7B, a small open-weights model, had high ICCs (≥0.88) but low Fleiss κ (0.32‐0.52). Few-shot+CoT prompting achieved the best concordance with human ratings for all the models (means across runs and rubric components), peaking for Mistral Large (accuracy 77%; Gwet AC1 and AC2 0.85, 95% CI 0.83‐0.87), followed by Claude (71%; 0.77, 0.74‐0.81), GPT-4o (67%; 0.72, 0.68‐0.77), and Mistral 7B (60%; 0.64, 0.56‐0.73). Concordance was higher for the binary rubric components (person and factual accuracy) than for the ternary components. Extreme deviations and invalid outputs were rare but more frequent in ternary components and the smaller local model.

**Conclusions:**

Large, general-purpose LLMs can function as reliable, rubric-guided raters of VP-based clinical summary statements, achieving high concordance with expert ratings under clearly defined criteria in binary rubric components and when using few-shot with CoT prompting. The performance on more nuanced, ternary reasoning dimensions was more variable. LLMs can support scalable, rubric-based formative feedback on clinical reasoning within VP systems but should complement rather than replace human judgment.

## Introduction

Clinical summary statements are a key element in physicians’ clinical reasoning. As concise, structured narratives, they facilitate transforming unstructured patient information into an initial diagnostic interpretation and considering relevant underlying differential diagnoses [1,2]. They make observable implicit parts of the reasoning processes that underpin diagnostic processes, and over time, they can be used to evidence the development of clinical reasoning competence. For example, rather than simply noting that “Mr. Miller came to the ER with several chest pain episodes during the last 2 hours,” a more advanced student would write, “Mr. Miller is a 62-year-old male with cardiovascular risk factors presenting with acute, substernal chest pressure radiating to the left arm, associated with diaphoresis, and relieved by rest.” This transformation from descriptive data to diagnostic framing exemplifies a key cognitive step in diagnostic reasoning [3]. However, evaluating the quality of these summary statements reliably and at scale remains a challenge in medical education, as many learners simultaneously need individualized, timely feedback.

Clinical summary statements are also referred to as problem representations or one-liners. Depending on the context, they can take various forms and serve different functions, such as a brief patient handover at the end of one’s shift or a more extensive case presentation at a clinical conference [4]. For this paper, we refer to clinical summary statements as extracts of patient information formulated by students during their learning process. Feblowitz et al [4] suggested a conceptual model that explains any clinical information summary as a sequence of 5 steps: (1) aggregation and (2) organization of clinical information, (3) reduction and transformation, (4) interpretation, and (5) synthesis. In educational research on summary statements, particular attention has been given to the last 3 steps of the sequence. Well-developed and effective summary statements include both positive and negative key findings, use semantic qualifiers (SQs) (eg, acute, radiating, and substernal), and thereby contextualize the presentation within epidemiological and demographic parameters; by doing so, they offer synthesized, essential case information within an abstracted, diagnostic framework [5,6]. Accordingly, assessing summary statements extends beyond assessing the factual correctness of the information presented by the learner. Rather, the evaluation should include how effectively learners structure information and attribute meaning to it. By sharing their interpretations and abstractions of patient information in summary statements, learners offer insight into how well they can integrate specific patient data into their illness scripts (ie, the knowledge of illnesses represented in their long-term memory [7]), the accuracy of their illness scripts, and how this process facilitates their overall clinical reasoning [1,8]. Diagnostic accuracy depends on these processes and can therefore be considered a summarizing indicator of clinical expertise [9].

Virtual patients (VPs) have become an established approach for medical students to learn clinical reasoning. These interactive, computer-based simulations allow them to train diagnostic processes in a safe, standardized, and scalable environment [10]. VPs enable repeated exposure to varied and even rare presentations and facilitate reflective, self-paced learning. Despite these advantages, providing students with reliable, individualized feedback on reasoning quality remains challenging. A recent review by Jay et al [11] revealed that among studies with learner feedback in VP systems, only a small fraction addressed feedback on the quality of clinical summary statements, and even fewer operationalized them as formative feedback for learning. This gap is relevant because feedback is a primary driver of learning in health professions education [12,13]. This points to a missed opportunity for medical students to learn in current educational approaches.

To address the gap in feedback provision, first VP studies with efforts to automate the evaluation of summary statements showed promising results: Hege et al [14] implemented a rule-based natural language processing (NLP) system grounded in a rubric by Smith et al [6], which assessed the following domains: SQ, transformation of information into higher-order concepts, diagnostic narrowing, factual accuracy, and overall quality in summary statements. Although the system achieved moderate agreement with medical expert ratings, extensive task-specific engineering, language-dependent dictionaries, and substantial training data were needed. Each new VP case demanded additional manual development, limiting scalability and adaptability. More fundamentally, rule-based systems sometimes struggled to capture the contextual nuances of clinical reasoning. Moreover, they can fail because of simple vocabulary mismatches (cf [15]), underscoring the need for approaches that interpret the meaning of summary statements rather than relying solely on pattern matching.

Recent advances in large language models (LLMs) offer a fundamentally different approach to the challenges encountered in many traditional rule-based NLP systems. LLMs process text through learned representations of semantic and contextual relationships, enabling nuanced interpretation without handcrafted rules [16,17]. LLMs also demonstrate better handling of inferences, the transformation of information, or different wording or phrasing that often fail with plain lexical lookups [18]. Their ability to generalize across content domains suggests the potential for scalable, context-aware evaluation of learner input. Nevertheless, the performance of LLMs depends on how the models are instructed to perform a task by prompting. Prompting refers to how instructions are structured to guide LLM behavior. Prompts are typically structured into (1) task instructions, (2) contextual information such as item stems or rubrics, (3) optional examples, and (4) an output specification. Well-thought-out prompt designs—particularly regarding the inclusion and phrasing of rubrics—have been shown to affect both the accuracy and reliability of automated scoring outcomes in medical education settings such as in Objective Structured Clinical Examination (OSCE) grading [19-21]. In zero-shot prompting, the model receives only task instructions (eg, “Score this text using the rubric and the scoring instructions below”) without examples. Few-shot prompting extends this by including a few examples of the task, allowing the model to infer the scoring logic from demonstration (eg, text examples paired with rubric scores given by raters). Chain-of-Thought (CoT) prompting, which adds a reasoning step to either few-shot or zero-shot prompting, can be combined with few-shot prompting to provide the LLM with human raters’ rationales for some exemplary human ratings (Methods section).

Despite emerging and promising evidence from automated assessments, no study has yet systematically examined the test-retest reliability of LLMs and their concordance with human expert ratings in evaluating clinical summary statements. Understanding whether LLMs can apply established rubrics is essential for determining whether using them for formative assessment in diagnostic-reasoning education is feasible. An LLM-based evaluation could help address the feedback gap identified by Jay et al [11] in current VP systems. Instead of relying on occasional, labor-intensive assessments by clinical teachers, learners could benefit from repeated VP-based practice, with LLMs providing immediate, consistent, expert-level feedback on their summary statements throughout medical training, thereby strengthening their clinical reasoning skills.

The aim of this study is to evaluate the performance of 4 transformer architecture–based, widely available LLMs with multilingual support in a rubric-guided evaluation of an existing set of clinical summary statements written by medical students during their interactions with online VPs.

Specifically, we formulate the following 2 research questions (RQs):

1. What is the test-retest reliability of LLM-generated medical summary statement ratings across 5 independent runs?
2. What is the concordance of LLM ratings with human expert ratings under different prompting configurations?

## Methods

### Study Design

Using an experimental comparative design, this study examined the test-retest reliability of LLMs in rubric-based ratings of medical summary statements under multiple prompting conditions and their concordance with human expert ratings.

Because LLM performance is highly dependent on the prompting techniques chosen [22], 4 prompting strategies (zero-shot and few-shot prompting, each with and without CoT) were applied. This resulted in a total of 16 experimental conditions across the 4 LLMs (4 prompting configurations × 4 LLMs). To better compare the results with those of the previous work on the automated evaluation of summary statements by Hege et al [14], the same summary statements from their study were used, as well as the evaluation rubric by Smith et al [6], which was annotated for LLM use (refer to the Rating Rubric and Prompt Development section).

Each of these combinations of models, prompt configurations (eg, zero-shot), and summary statements was run 5 times.

### Models and Infrastructure

Four off-the-shelf general-purpose LLMs were tested: 3 large commercial models (Anthropic’s Claude Sonnet 4, OpenAI’s GPT-4o, and Mistral AI’s Mistral Large) and one smaller local model (Mistral AI’s Mistral 7B). The models were selected purposefully to cover the range of deployment options at the time of the study: 3 widely used high-performing commercial models from different vendors and a smaller open-weight model that can be deployed locally. Specialized domain-specific models for medical purposes, such as Google’s Med-PaLM [23] and MedAlpaca [24], or BERT (Bidirectional Encoder Representations from Transformers)-like models, such as MedBERT [25], exist. However, these models are currently either less accessible to the public and/or not mature or stable enough for reliable or automated assessment, which were 2 key research aspects of this work; thus, these specialized models were not included in our study. The local Mistral 7B model was obtained from Hugging Face, a community-driven website that shares language models and tools. We used an activation-aware weight quantization (AWQ) variant of Mistral 7B, a compressed version that is optimized for lower hardware profiles while still keeping its core features [26].

An overview of the models and specifications is provided in Table 1.

**Table 1.** Overview of the various LLMs^a^ that were used and compared in our study.

Provider	Model	Exact version
Anthropic	Claude Sonnet 4	claude-sonnet-4‐20250514
OpenAI	GPT-4o	gpt-4o-2024-05-13
Mistral AI	Mistral Large	mistral-large-2411
Mistral AI	Mistral 7B	TheBloke/Mistral-7B-Instruct-v0.2-AWQ (via Hugging Face)

a LLMs: large language models.

### Data Source

The dataset from Hege et al [14] comprising 125 rubric-scored summary statements in English (n=84) and German (n=41) was used for this study. The summary statements were written by medical students in response to 6 human-written virtual patient cases in internal medicine from open-access clinical reasoning courses in Instruct’s CASUS [27] VP system. They varied in length and quality, ranging from near-perfect to incomplete summaries. In a study by Hege et al [14], the statements were scored independently by 2 health care professionals with an educational background and expertise in clinical reasoning. The ratings of the statements were based on an elaborated rubric by Smith et al [6]. To calibrate their use of the rubric, the 2 experts independently rated and discussed 25 sample summaries before rating the full set of 125. Divergent scores were discussed and rating consensus was reached for all summary statements [14].

For this study, 3 of the 125 summary statements available were excluded from the current analysis because they were used as examples in few-shot prompting conditions, leaving 122 statements for evaluation.

### Rating Rubric and Prompt Development

For systematic evaluation, we applied the rubric developed by Smith et al [6] as adapted by Hege et al [14], an established assessment tool with prevalidated quantitative scoring scales, consisting of binary and ternary items. This instrument was used directly as our rating standard and for developing corresponding prompt instructions. The rubric comprises 6 criteria (components): (1) SQ, (2) transformation of information, (3) narrowing of differential diagnosis, (4) factual accuracy, (5) patient name or person (added by Hege et al [14]), and (6) a global score (a separate, subjective overall score). The rubric components no. 4 and 5 were binary (scoring: 0/1), whereas the rest were ternary (with 3 possible scorings: 0, 1, or 2).

Four prompting strategies were implemented and assessed, following established LLM practices from the prompting literature [19,22,28]:

1. Zero-shot, no CoT
2. Zero-shot with CoT
3. Few-shot, no CoT
4. Few-shot with CoT

Zero-shot prompting relied on the model’s pretrained knowledge without rating examples, whereas in few-shot prompting, we included 3 manually picked summary statements of our dataset and annotated them with human expert ratings, to serve as rating examples representative for our dataset. They were chosen specifically to exemplify all possible proficiency levels (0/1 in binary and 0/1/2 in ternary rubric components) across 3 different VP cases: 2 in English and 1 in German. CoT prompting encouraged stepwise reasoning by appending the phrase “Let’s think step by step” (zero-shot CoT). In few-shot prompting, CoT was additionally supported by guided step-by-step annotations on how to score provided examples [19]. On the basis of the prompting condition, different prompt segments were included, as summarized in Table 2. The exact prompt texts in Markdown format are provided in Multimedia Appendix 1. All additional data, such as the exact case vignettes, can be found in our GitHub repository (Data Availability section).

**Table 2.** Composition of each prompt depending on the prompting condition. The prompts contained different prompt segments.

Prompt segment	Zero-shot	Zero-shot CoT^a^	Few-shot	Few-shot CoT
Role description	✓^b^	✓	✓	✓
Scoring rubric	✓	✓	✓	✓
VP^c^ case	✓	✓	✓	✓
Three scoring examples	◦	◦^d^	✓	✓
Case information for exemplary human case scorings	◦	◦	✓	✓
Rationales behind examples	◦	◦	◦	✓
Student response placeholder where summary statement was inserted	✓	✓	✓	✓
CoT initiator	◦	✓	◦	✓

a CoT: chain-of-thought.

b ✓ denotes a segment included in prompt

c VP: virtual patient.

d ◦ denotes segment not included in prompt.

The prompts were iteratively refined in the process as described by Lee et al [19]:

The rubric content and assessment rationales were formatted as a prompt and then reviewed by the study authors, including experts in educational research and AI (PB, PS, and IH). This resulted in a more detailed description of SQ based on Chang et al [1] and minor linguistic changes (eg, case vignettes → clinical cases). For the initial iterative test runs, the LLMs were prompted to provide explanatory ratings (ie, rationales) to identify cardinal ambiguities, which led to further prompt refinement (PB, PS). This optimization was performed mainly with Mistral 7B, the smallest model (ie, fewest parameters) in our study, to ensure robustness across models, under the assumption that larger models would properly process prompts modified to work with the smallest model [29]. Explanations provided by LLMs generally matched given scores, eg, when the patient name was scored with 1, the model would provide an adequate justification that the statement included the patient name. During this step, we identified that insufficient formatting led Mistral 7B and Claude Sonnet 4 to systematically confuse VP cases with the summary statement to be rated, which resulted in increased use of special characters to provide a clear structure in crucial areas of the prompt (such as the use of brackets and delimiters). Based on the ratings of summary statements excluded from the analysis, we refined the description of the scoring rubric (PB, PS, and JJK). Lastly, the final step of prompt development was to instruct all models to solely output the raw scores without further rationales.

A comparative overview of the original scoring rubric and the interpretative information and rationales that we added to minimize ambiguity for LLM interpretation are presented in Multimedia Appendix 2.

### Procedure

The experiments were conducted between July and September 2025 using the latest available models at that time. The 5 runs per model and prompting condition were executed consecutively, and a new run automatically started as soon as the previous run completed, resulting in a narrow time window of around 1 to 2 hours in which the data were collected per experiment condition, depending on the model and prompting condition.

The local Mistral 7B model was deployed using vLLM [30] and was run on a single NVIDIA Tesla T4 GPU via our institution’s compute cloud. All other models (Claude, GPT-4o, and Mistral Large) were accessed via their official APIs using the same LangChain setup to ensure comparability between the commercial online models and the local model. No conversation memory was implemented, preventing learning effects between reruns of ratings. Each of the 122 summary statements was rated under all 16 experimental conditions across 6 rubric components, which was repeated 5 times, yielding a total of 58,560 data points collected as scores across all 6 rubric components.

We implemented custom software to automate the process using the Python programming language (Python Software Foundation) with the LangChain v0.3.26 package, a software abstraction that handles LLM inference. For each model vendor, we used the respective LangChain submodule to access the APIs. To ensure data integrity, we formulated a strict data format and passed each LLM call to a structured output function that automatically ensures that the LLMs’ responses follow that format. Our custom software procedure included (1) resolving all placeholders in the prompt with the data for each individual run (the summary statement to be rated and the virtual patient case vignette; 3 examples for few-shot conditions; annotations for the examples, and a CoT reasoning initiator for CoT conditions), (2) sending the complete prompt to the LLM API via LangChain, (3) obtaining the raw results from the LLM API, (4) transforming the data into a structured format, and (5) adding the data to a CSV file for later statistical analysis. Temperature, a model-specific hyperparameter, was set to zero for all models for more deterministic results. It should be noted, though, that even a temperature of zero does not guarantee fully deterministic results when working with closed APIs [31]. This is also captured further in the Results section (see RQ1 for test-retest reliability), where we report on the test-retest reliability between consecutive runs.

Our GitHub repository includes the exact code, prompts, scoring materials, and original human reference ratings to replicate the study (see Data Availability section).

### Data Analysis

Statistical analysis was performed using R v4.5.0 [32]. The complete R and Python codes used for this study can be found in our GitHub repository (see the Data Availability section).

We analyzed the resulting dataset from 2 complementary perspectives.

To address RQ1, we assessed test-retest reliability by examining whether the models produced consistent and stable ratings across the 5 run repetitions, measured as (1) consistency using intraclass correlation coefficients (ICCs) and (2) intrarater agreement using Fleiss κ (effectively treating each run as an independent measurement by the same LLM rater). The ICC was calculated using a one-way random-effects model based on the average of 5 raters according to [33]. By examining the consistency of the 5 aggregated runs, this approach was chosen to examine whether random model variances affect a model’s scoring behavior, even when the API temperature was set to zero. The ICC is used to capture consistency in numeric scoring, whereas Fleiss κ is designed for exact categorical agreement. Both complement each other well in our setup: the meaning of score distances is not entirely uniform across rubric components (eg, a change from 0 to 1 does not necessarily equal a change from 1 to 2); thus, both numeric consistency and categorical agreement perspectives provide information on the degree of systematic bias.

To address RQ2, we assessed concordance with human expert ratings using 2 metrics: (1) accuracy (percentage of exact LLM-human match agreement) and (2) interrater agreement measured using Gwet AC1 and AC2 coefficients (chance-corrected indices) for comparison with prior work. Following the recommendations for robust agreement assessment, we prioritized Gwet AC1 and AC2 as our primary statistic, as suggested by Xu and Lorber [34]. AC1 is a suitable metric for binary (categorical) rubric components, whereas AC2 provides a measure for ordinal components, in our case, ternary rubric components [35,36]. Conventional indices such as Cohen kappa and Krippendorff α can underestimate agreement in cases where the distribution of ratings is imbalanced (eg, when most statements are factually correct, as in our dataset). This prevalence problem is well documented and can lead to artificially low reliability scores despite strong observed agreement. Gwet AC1 and AC2 correct for this bias [35,37] and are therefore considered more robust for skewed datasets [38,39]. For each combination of model, prompting configuration, and rubric component, Gwet AC1 and AC2 and weighted Cohen kappa coefficients were calculated separately for each of the 5 runs. For results per rubric component, we report the mean, SD, and SE across these 5 runs. For overall results per model and prompting configuration, we report the mean, SD, and SE across all 30 coefficients (6 rubric components×5 runs). The 95% CIs were calculated using a Wald-type normal approximation (mean±1.96×SE).

In accordance with prior research, we applied the Landis and Koch scale [40] to interpret the raw Gwet AC1 and AC2 values. However, this interpretation needs to be treated with some care, as Gwet AC1 and AC2 tend to produce higher values than Cohen kappa does [37]. To allow a full comparison between all the models, prompting conditions, and rubric components, we included the full values of Gwet AC1 and AC2 and weighted Cohen kappa–for a more complete comparison with related work–over all 5 runs in Multimedia Appendices 3 and 4.

### Ethical Considerations

The secondary data analysis conducted in this study was approved by the Ethics Committee of the Ludwig-Maximilians-Universität München, Germany (reference number 260–15), with the primary data having been generated by Hege et al [14]. An additional informed consent was not required for the anonymous analysis of the data. The virtual patients provided to the learners were fictitious and not based on real individuals.

## Results

### RQ1: Test-Retest Reliability

The ICCs indicate high consistency across 5 repeated runs for all LLMs (ICC*≥*0.88), with varying levels of intrarater agreement depending on the model and prompting condition (Fleiss κ=0.32‐1.00), as shown in Table 3.

Figure 1 shows the deviations between model and human ratings, as well as the number of invalid outputs (eg, numerical ratings outside the rubric scale), for the first of the 5 runs. The maximum possible deviation was ±2 points for ternary components (0-2 scale) and ±1 point for binary components (0-1 scale). Enlarged versions of the panels of Figure 1 can be found in the Multimedia Appendices 5-8.

**Table 3.** ICC^a^ and Fleiss κ for all the model × prompt configurations. Claude was fully stable across all runs, whereas Mistral 7B had weaker Fleiss κ values despite a high ICC.

Configuration	ICC	Fleiss κ
Mistral Large zero-shot	0.92	0.66
Mistral Large few-shot	0.97	0.80
Mistral Large zero-shot CoT^b^	0.98	0.91
Mistral Large few-shot CoT	0.97	0.80
Claude zero-shot	1.00	1.00
Claude few-shot	0.99	0.99
Claude zero-shot CoT	0.99	0.99
Claude few-shot CoT	0.99	0.99
GPT-4o zero-shot	0.92	0.59
GPT-4o few-shot	0.99	0.94
GPT-4o zero-shot CoT	0.99	0.94
GPT-4o few-shot CoT	0.99	0.92
Mistral 7B zero-shot	0.88	0.32
Mistral 7B few-shot	0.91	0.43
Mistral 7B zero-shot CoT	0.89	0.39
Mistral 7B few-shot CoT	0.92	0.52

a ICC: intraclass correlation coefficient.

b CoT: chain-of-thought.

**Figure 1.**
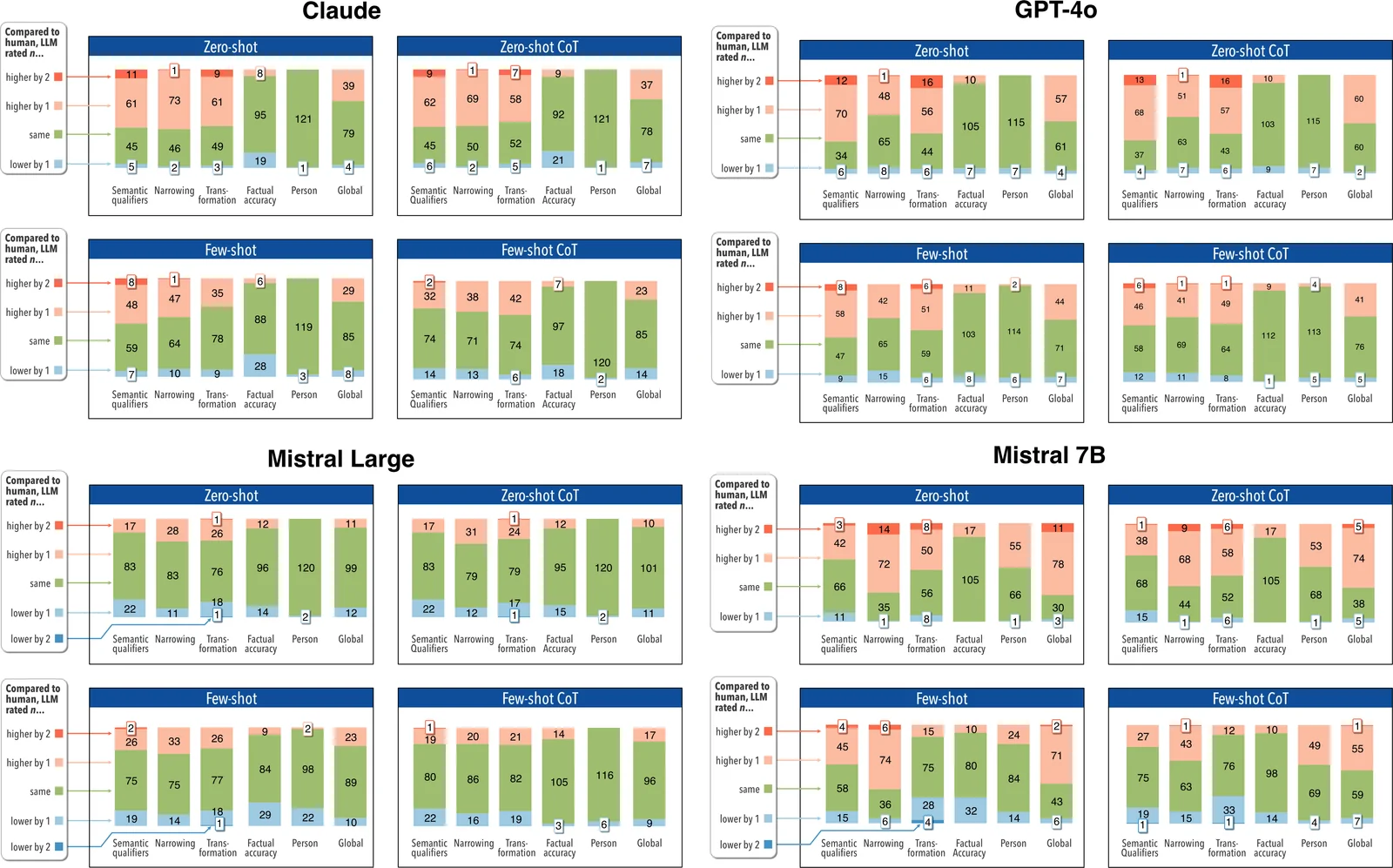
Predicted large language model ratings per model, prompting configuration, and rubric component. The stacked bars show the absolute number of equal (same) ratings, as well as ratings that were lower by 1 or 2 or higher by 1 or 2 compared with the human reference. The results of the first of the 5 runs per model and configuration were used for this figure. We have included enlarged versions of the individual panels from this figure in Multimedia Appendices 5-8 to make them easier to read. CoT: chain-of-thought; LLM: large language model.

Claude was the most reliable: zero-shot runs were essentially deterministic (ICC=1.00*,* Fleiss κ=1.00)*,* whereas all other conditions were near-deterministic (ICC≥0.99, Fleiss κ≥0.99)*.* For the other models, zero-shot runs yielded the lowest ICC and Fleiss κ values. GPT-4o (excluding zero-shot) achieved very high test-retest reliability (ICC≥0.99 and Fleiss κ≥0.92)*.* Mistral Large (also excluding zero-shot) showed lower values but nevertheless high consistency and intrarater agreement (ICC≥0.97, Fleiss κ≥0.80)*.*

In zero-shot settings, both GPT-4o and Mistral Large showed high consistency (ICC=0.92) and moderate to substantial intrarater agreement (Fleiss κ=0.59 and =0.66, respectively). The lowest values were reported for Mistral 7B, with high ICC (≥0.88) but only fair-to-moderate intrarater agreement (Fleiss κ=0.32‐0.52).

### RQ2: Concordance of LLM Ratings With Human Experts—Interrater Agreement (Gwet AC1 and AC2)

In our study, no single LLM-prompt configuration achieved superior agreement across all the rubric criteria. Figure 2 visualizes Gwet AC1 and AC2 by model, rubric, and prompting techniques. While simple zero-shot prompting achieved adequate agreement on specific rubric components, it failed to perform consistently across the entire rubric. Supplementing models with few-shot examples and/or CoT prompting was required to achieve consistent, substantial to almost-perfect agreement across most rubric components.

**Figure 2.**
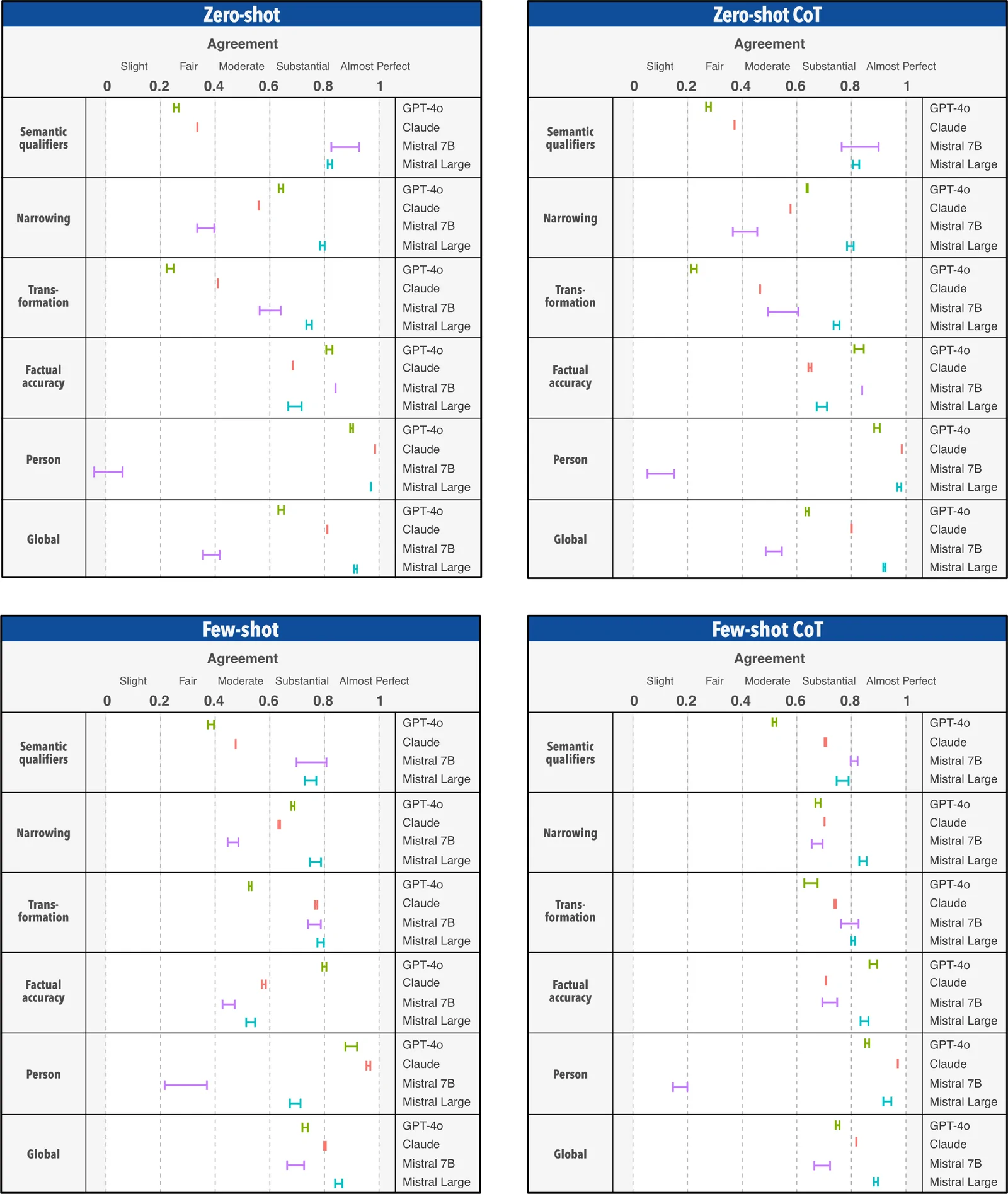
Agreement values according to Gwet AC1 and AC2 by rubric component, prompting configuration, and model. Binary components (person, factual accuracy) generally reached higher reliability than abstract ternary components (semantic qualifiers, transformation, narrowing, and global). Agreement values are interpreted using the Landis and Koch scale [40], which ranges from slight to almost perfect agreement. CoT: chain-of-thought.

To comprehensively evaluate how LLM selection, prompting configuration, and the rubric component impact performance, we conducted a linear mixed-effects model followed by a Type III ANOVA using R’s *lme4* and *lmerTest* packages. To control for the nonindependence of repeated runs, we included the unique identifier for each individual batch (combining model, prompt configuration, and run index) as a random intercept. Satterthwaite method was subsequently applied to approximate the denominator degrees of freedom for the fixed effects. The analysis evaluated run-level Gwet AC1 and AC2 coefficients across all 5 runs per experimental condition. The analysis revealed significant main effects for model (*F*_3, 64_= 2178.01, *P*<.001), prompting configuration (*F*_3,64_=538.21, *P*<.001), and rubric component (*F*_5,320_=714.33, *P*<.001). Crucially, all 2-way and 3-way interaction effects were also significant (*P*<.001), most notably the model × rubric component interaction (*F*_15,320_=1717.72, *P*<.001). This indicates that while model choice and prompt configuration dictate overall performance, their efficacy is deeply tied to the specific nature of the rubric component being evaluated.

### Differences by Model and Prompting Configuration

The concordance with human experts varied greatly depending on the selected model and even more for the prompt configuration. The mean Gwet AC1 and AC2 coefficients per model and prompting configuration are reported in Table 4.

**Table 4.** Gwet AC1 and AC2 interrater agreement coefficients for the 4 LLMs^a^ across 4 prompting configurations (zero-shot and few-shot, with or without CoT^b^). For each model and prompting configuration, Gwet AC1 (binary components) or AC2 (ternary components) was calculated separately for each of the 6 rubric components in each of the 5 runs. The table shows the mean, SD, SE, and 95% CI of these 30 coefficients. The SD, SE, and 95% CI mainly reflect differences in agreement between rubric components.

Model and configuration	Mean (SD)^c^	SE^c^	95% CI^c^	
Claude				
Few-shot	0.70 (0.16)	0.03	0.64-0.76	
Few-shot CoT	0.77 (0.10)	0.02	0.74-0.81	
Zero-shot	0.63 (0.23)	0.04	0.54-0.72	
Zero-shot CoT	0.64 (0.21)	0.04	0.56-0.72	
GPT-4o				
Few-shot	0.67 (0.17)	0.03	0.61-0.74	
Few-shot CoT	0.72 (0.13)	0.02	0.68-0.77	
Zero-shot	0.58 (0.26)	0.05	0.49-0.68	
Zero-shot CoT	0.58 (0.26)	0.05	0.49-0.68	
Mistral Large				
Few-shot	0.73 (0.10)	0.02	0.69-0.77	
Few-shot CoT	0.85 (0.06)	0.01	0.83-0.87	
Zero-shot	0.82 (0.10)	0.02	0.79-0.86	
Zero-shot CoT	0.82 (0.10)	0.02	0.79-0.86	
Mistral 7B				
Few-shot	0.57 (0.19)	0.03	0.5-0.64	
Few-shot CoT	0.64 (0.22)	0.04	0.56-0.73	
Zero-shot	0.52 (0.30)	0.05	0.41-0.63	
Zero-shot CoT	0.54 (0.26)	0.05	0.44-0.64	

a LLM: large language model.

b CoT: chain-of-thought.

c Gwet AC1 and AC2.﻿

Mistral Large showed the best overall performance. Under optimal prompting, it reached almost perfect agreement on Person (AC1=0.976, 95% CI 0.969‐0.983 in zero-shot CoT), Global (AC2=0.921, 95% CI 0.917‐0.925 in zero-shot CoT), Narrowing (AC2=0.842, 95% CI 0.829‐0.855 in few-shot CoT), SQ (AC2=0.820, 95% CI 0.811‐0.828 in zero-shot), and transformation (AC2=0.807, 95% CI 0.800‐0.813 in few-shot CoT). It was less effective for the binary component factual accuracy, with the lowest agreement in few-shot (AC1=0.530, 95% CI 0.514‐0.546), but under few-shot CoT, it increased to 0.848 (95% CI 0.833‐0.862). Interestingly, compared with zero-shot prompting, few-shot prompting reduced agreement, but this effect was mitigated when few-shot prompting was supplemented with CoT.

GPT-4o excelled in the binary rubric components, with factual accuracy peaking at AC1=0.880 (95% CI 0.866‐0.894) in few-shot CoT and consistently high agreement on Person (lowest AC1=0.858, 95% CI 0.850‐0.865 in few-shot CoT, highest 0.899, 95% CI 0.894‐0.904 in zero-shot). However, it struggled with 2 of the 4 ternary categories in zero-shot: SQ (AC2=0.257, 95% CI 0.248‐0.266) and transformation (AC2=0.235, 95% CI 0.222‐0.247), corresponding to fair agreement. Only the combination of few-shot and CoT markedly improved these components (AC2=0.518, 95% CI 0.511‐0.525, and AC2=0.652, 95% CI 0.627‐0.676, respectively). Notably, under zero-shot, CoT had little effect across the whole rubric.

Claude reached the highest agreement in person across all prompting techniques (lowest AC1=0.961, 95% CI 0.954‐0.968 in few-shot CoT; highest AC1*=*0.985, 95% CI 0.985‐0.985 in both zero-shot and zero-shot CoT). It also achieved almost perfect agreement globally across all prompting conditions (lowest AC2=0.801, 95% CI 0.801‐0.801 in zero-shot CoT; highest AC2=0.818, 95% CI 0.818‐0.818 in few-shot CoT). In the other ternary categories, Claude reached fair to moderate agreement in zero-shot; however, a combination of few-shot and CoT yielded substantial gains in agreement: SQ increased from AC2=0.334 (95% CI 0.334‐0.334) to 0.705 (95% CI 0.702‐0.708), Transformation from 0.409 (95% CI 0.409‐0.409) to 0.740 (95% CI 0.738‐0.743) and narrowing from 0.559 (95% CI 0.559‐0.559) to 0.701 (95% CI 0.701‐0.701). CoT in zero-shot, as in GPT-4o, seemed ineffective except for minimal gains in the rubric component person.

Mistral 7B Local showed the greatest variance among all the tested models. In zero-shot, it performed well in SQ, achieving almost perfect agreement (AC2=0.876; 95% CI 0.825‐0.927); however, at the same time, person collapsed to near chance (AC1=0.037; 95% CI –0.016 to 0.089). Prompting did not improve Mistral 7B’s performance in person. Factual accuracy was highly dependent on the configuration: weak in few-shot (AC1=0.449, 95% CI 0.427‐0.472) but strong in zero-shot and zero-shot CoT (in both AC1=0.840, 95% CI 0.840‐0.840). Narrowing and transformation benefitted from the combination of few-shot and CoT, reaching AC2=0.675 (95% CI 0.655‐0.694) and 0.794 (95% CI 0.762‐0.826), respectively. In Global, few-shot improved agreement independently of the presence of CoT.

### Differences by Rubric Component

The binary components usually showed the highest agreement among the rubric components. The best agreement on person was achieved in Claude zero-shot and zero-shot CoT (both ex aequo with deterministic output at AC1=0.985, 95% CI 0.985‐0.985), followed closely by Mistral Large under zero-shot CoT prompting (AC1=0.976, 95% CI 0.969‐0.983). Factual Accuracy peaked with GPT-4o in few-shot CoT (AC1=0.880, 95% CI 0.866‐0.894). Notably, Mistral 7B Local struggled in the person category and produced outliers, failing to achieve even moderate agreement across all prompting strategies (AC1≤0.292). Ternary rubric components led to more variable results. Global ratings were best with Mistral Large in zero-shot CoT (AC2=0.921, 95% CI 0.917‐0.925); however, in terms of this component, Claude also consistently reached almost perfect agreement (the lowest AC2=0.801, 95% CI 0.801‐0.801). For narrowing, Mistral Large few-shot CoT performed best (AC2=0.842, 95% CI 0.829‐0.855). The agreement on the SQ was high among both Mistral models, with Mistral 7B Local in zero-shot reaching the highest AC2=0.876 (95% CI 0.825‐0.927). Transformation was most reliably scored by Mistral Large in few-shot CoT, with AC2=0.807 (95% CI 0.800*‐*0.813)*.* Interestingly, for most models, transformation consistently benefited from few-shot prompting but not from CoT. We summarize the best model and prompting configuration per rubric component in Table 5.

**Table 5.** Highest interrater agreement by rubric component. The best model × prompt configuration per rubric component was selected according to Gwet AC1 in binary and AC2 in ternary rubric components. Claude showed the same performance in zero-shot with and without CoT^a^ in the person rubric category.

Rubric component	Model	Configuration	Mean (SD)	SE	95% CI	
Binary (AC1)						
Factual accuracy	GPT-4o	Few-shot CoT	0.880 (0.016)	0.007	0.866-0.894	
Person	Claude	Zero-shot CoT	0.985 (0.000)	0.000	0.985-0.985	
Person	Claude	Zero-shot	0.985 (0.000)	0.000	0.985-0.985	
Ternary (AC2)						
SQs^b^	Mistral 7B	Zero-shot	0.876 (0.058)	0.026	0.825-0.927	
Narrowing	Mistral Large	Few-shot CoT	0.842 (0.016)	0.007	0.829-0.855	
Transformation	Mistral Large	Few-shot CoT	0.807 (0.007)	0.003	0.800-0.813	
Global	Mistral Large	Zero-shot CoT	0.921 (0.004)	0.002	0.917-0.925	

a CoT: chain-of-thought.

b SQs: semantic qualifiers.

### Exact-Match Agreement (Accuracy) and Absolute Deviations From Human Experts

The few-shot with CoT prompting technique achieved the highest overall accuracy across all the models (Figure 3). Accuracy was calculated as the percentage of LLM ratings that exactly matched the human reference rating, averaged over all summary statements and rubric components. Peak values were observed for Mistral Large, where few-shot+CoT achieved 77% accuracy. The second-highest accuracy (76%) was achieved by Mistral Large zero-shot+CoT and Mistral Large zero-shot.

**Figure 3.**
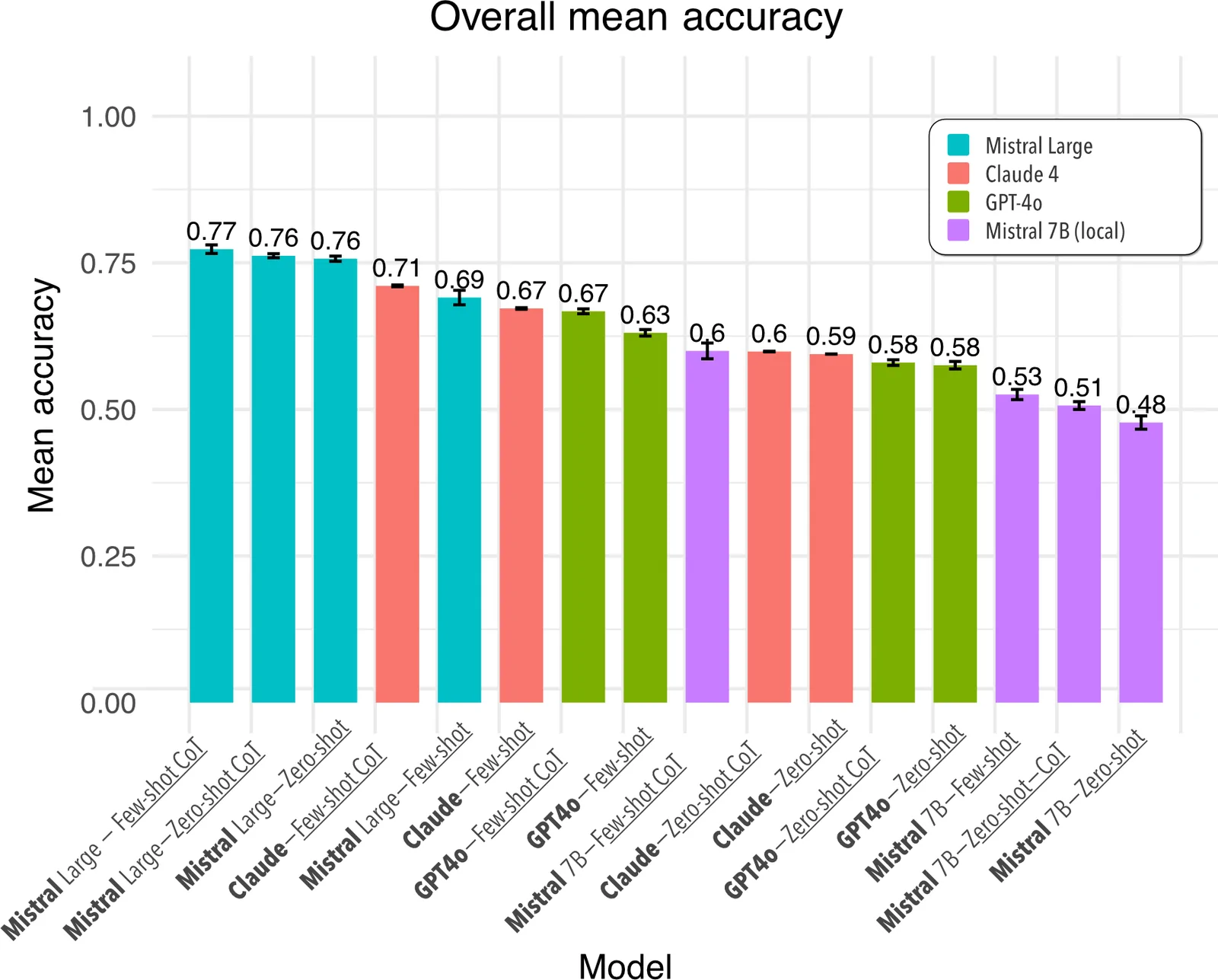
Overall mean accuracy (exact-match agreement with human expert ratings) per model and prompting configuration. Error bars show SDs across the 5 runs. CoT: chain-of-thought.

In few-shot with CoT, Claude achieved 71% accuracy, and GPT-4o reached 67%. Mistral 7B achieved 60% accuracy in few-shot CoT. It was higher than Claude in the zero-shot condition (59%) and GPT-4o in both the zero-shot and zero-shot+CoT settings (58%). Mistral 7B in zero-shot+CoT (51%) and zero-shot (48%) showed the lowest accuracy.

Deviations from human ratings were chosen as additional robustness checks. Extreme deviations of ±2 points from the human reference ratings were rare. Mistral Large showed the fewest such deviations, whereas Claude, GPT-4o, and Mistral 7B occasionally produced ±2 deviations, particularly in the SQs and Transformation categories (Figure 1). Rarely, invalid outputs were observed as described in the next section.

### Handling Invalid Outputs

A total of 8 invalid outputs were observed (ie, scores outside the rubric scale, such as a 3 on a 0/1/2 scale, or a 2 on a binary rubric component) that only occurred with Mistral 7B as summarized in Table 6. The invalid outputs were found in all prompting configurations but few-shot with CoT. We excluded from all further analysis every summary statement entry in which an invalid data point occurred. Due to the very small number of excluded data points, their impact on reliability and agreement estimates is negligible.

**Table 6.** Overview of excluded invalid values from analysis. Initial sample size: 122 summary statements×5 runs (610 entries per configuration).

Configuration	Initial sample size	Number of excluded entries	Final analyzed sample size
Mistral 7B few-shot	610	1	609
Mistral 7B zero-shot	610	5	605
Mistral 7B zero-shot with CoT^a^	610	2	608

a CoT: chain-of-thought.

## Discussion

### Principal Findings

The primary aim of this study was to examine whether and to what extent LLMs can rate medical students’ written summary statements from VP cases using a rubric and thereby help address the lack of individualized feedback in most VP systems. Our findings indicate that LLMs can serve as robust raters, providing a basis for scalable, automated feedback on summary statements in VP-based training. However, this requires careful consideration, as consistency and concordance with human experts depend on several factors: model choice, prompting configuration, temperature setting, and the rubric itself. There is no single best configuration; educators need to evaluate iteratively which combination of model and prompt works for their setting.

Across 5 repeated runs per prompting configuration, Mistral Large, Claude Sonnet, and GPT-4o produced stable scores with high test-retest reliability and substantial to almost perfect agreement with human experts, whereas the smaller local model Mistral 7B showed less consistent behavior across rubric components and prompting configurations. Few-shot with CoT prompting yielded the highest overall concordance with expert human raters, with Mistral Large performing best and Claude and GPT-4o also benefiting from illustrative examples and explicit reasoning initiators.

Divergences between high ICC values and lower Fleiss kappa values suggest a systematic scoring bias but highlight random stability, which could be due to an offset in the models’ internal scoring scale that may be anchored differently from the human expert scale. This pattern was most frequently observed in GPT-4o and Mistral Large under zero-shot settings. Such systematic bias has practical implications for formative assessment, where absolute scores matter for student feedback, and argues for score calibration or normalization steps when using LLMs to score medical summary statements.

Rubric components displayed distinct performance patterns. Binary criteria (person and factual accuracy) generally achieved higher agreement than ternary criteria did. This could indicate that current LLMs capture surface-level correctness and easily operationalized features more reliably than they capture more nuanced aspects of clinical reasoning. However, this discrepancy might also stem from the frequency distribution of ratings within the human reference dataset (Multimedia Appendix 9). Future work should further investigate how rating distributions in reference datasets influence these results. To characterize agreement with the human reference standard, we prioritized Gwet AC1 and AC2, which reduced the underestimation of agreement observed with conventional coefficients such as Cohen kappa in skewed rating distributions.

### Comparison With Prior Work

Recent work on automated scoring has shown that LLMs can approach or match human raters in terms of reliability and interrater agreement across a range of educational contexts. Seßler et al [41] reported high intrarater reliability (ICC up to 0.84 for closed-source models) and strong alignment with teacher ratings in multidimensional essay scoring [41], whereas Hackl et al [42] reported exceptionally high consistency for GPT-4 (ICC=0.94‐0.99) in rating short written responses with minimal scoring drift under deterministic parameters. In medical education, Runyon [20] demonstrated that GPT-4o and Claude can achieve human-level accuracy when analytic rubrics are applied to postencounter notes if the prompts and scoring instructions are carefully engineered. Complementary studies by Alshammari et al [43] and Sreedhar et al [44] showed that LLMs can reach accuracy and consistency sufficient for formative use in answer-based and critical appraisal tasks, respectively. Our finding that large LLMs yield high ICCs and AC1 and AC2 values, particularly when well prompted, aligns with these findings that LLMs can act as reliable raters for structured, rubric-based assessments.

Integrating CoT reasoning with explicit rubric information improves both scoring accuracy and interpretability [19,45], but it is cautioned that simple zero-shot prompting tends to produce systematic overrating and weaker alignment with human judgments in content-rich or logic-heavy tasks [41,46]. In medical education, Runyon [20] and Jamieson et al [20,21] reported that prompts that embed analytic rubrics and provide examples enable LLMs to score postencounter notes with reliability comparable to that of faculty while dramatically reducing grading time. Similarly, Sreedhar et al [44] reported that rubric-guided prompting yields substantial agreement with faculty ratings. Our results replicate and extend these observations to VP-based summary statements: for all the models, few-shot with CoT prompting produced the highest agreement with human ratings, and no single LLM-prompt configuration dominated across all the rubric components. This finding supports the idea that prompt design and rubric operationalization are at least as important as model choice for achieving reliable scoring.

Our study also supports prior work emphasizing that LLMs perform best on clearly defined criteria and more variably on abstract reasoning dimensions. Across the models, the agreement was systematically greater for binary rubric components than for ternary ones. This pattern is consistent with views of clinical reasoning as a context-dependent, knowledge-intensive process that integrates multiple information sources [47]. It also resonates with findings that while LLMs handle pattern-based or shallow tasks well, they may struggle with more complex, compositional reasoning [48]. In our data, components such as transformation and narrowing benefited from few-shot with CoT prompting. Nevertheless, they did not reach the same level of robustness as binary criteria did, indicating that the more abstract the rubric dimension is, the more fragile automated scoring becomes.

The black-box character of LLMs further complicates their use as stand-alone graders. Because these models rely on learned internal representations rather than explicit symbolic rules, reconstructing exactly why a given score was assigned is difficult [48]. Runyon [20] proposed separating analytic explanation from score assignment, prompting the model first to analyze an answer and only then to derive a score to reduce complexity and improve transparency. Our findings support this line of argument: occasional extreme deviations and rare invalid outputs in our robustness checks, particularly for composite or global scores, indicate that models can still fail in ways that are hard to interpret and justify to learners.

Finally, the educational impact of LLM-based feedback must be considered in light of how learners experience it. Ali et al [49] reported that students value the immediacy of AI-generated feedback but still rate human tutor feedback higher in terms of clarity and usefulness. This aligns with the broader concept of hybrid intelligence, where human and machine capabilities are combined to compensate for each other’s weaknesses [50]. Our results fit well within this framework: LLMs appear capable of providing frequent, structured ratings of VP summary statements that could meaningfully expand feedback provision.

### Limitations

First, we used a single combined reference rating from 2 human expert evaluators. Although predefined criteria and adjudicated disagreements supported consistent, context-appropriate judgments, this approach limits rater diversity and may not fully capture the variability of expert opinion. Future research should therefore involve a larger pool of raters, report interrater reliability between humans explicitly, and use these data as an additional benchmark for comparing LLM performance.

Second, the options for analyzing and comparing test-retest reliability across different LLMs (RQ1) depend statistically on the scale level of the assessment rubric. In the rubric used for this study, several components have ordinal intervals that do not represent equal distances, so the parametric ICC should be interpreted with caution. We therefore added Fleiss kappa as an exact categorical agreement metric, which served as our primary benchmark for test-retest reliability per LLM, while the ICC provides complementary information on the degree of systematic bias.

Third, the dataset comprised summary statements from selected VP cases in internal medicine only. It remains unclear whether our findings can be generalized to other disciplines, to different VP designs, or to more complex cases. Additional prompt refinement—such as further clarifying rubric descriptions or adding targeted examples—might be necessary when this approach is transferred to new contexts as LLM-generated rationales for rubric-based ratings depend on prompt wording during prompt development (for details, see Methods section). In our study, an effective prompting technique was few-shot with CoT. It included an LLM role description, the scoring rubric, the VP case context, scoring examples with corresponding cases, step-by-step rationales for those examples, an explicit CoT initiator, and a placeholder for the learner’s summary statement. Future studies should systematically examine which of these elements are essential, how many examples are optimal, and whether strategies such as selecting extreme, typical, or atypical examples for few-shot prompting can further improve the scoring quality.

Fourth, our dataset contained virtual patient case vignettes and summary statements in English and German. Since the German sample size (n=41) was relatively small, we did not stratify our evaluation metrics by language to avoid unstable statistical estimates. Comparisons between common LLMs indicate that the performance for many high-resource languages (HRLs), such as English and German, is practically equal [51]. Future research with larger, balanced multilingual samples is needed to determine whether system performance varies significantly by language.

Fifth, regarding our model selection, new models such as GPT-5 and Claude Sonnet 4.5 became available after we completed data collection and were therefore not included. While their performance may differ, we argue that careful prompt design and robust evaluation strategies are more critical than any specific model choice for this type of task. Moreover, we deliberately restricted our analysis to baseline (ie, nonreasoning) LLMs and did not include large reasoning models (LRMs), such as OpenAI’s o1 or Claude Sonnet 4.5 Thinking. This decision was made to maintain a consistent and comparable study design. Reasoning models introduce additional training stages and inference steps that may add uncertainty and require new evaluation frameworks [52,53]. Our goal here was to provide comprehensive evidence on commonly available baseline models as a first step. Future studies should systematically investigate how LRMs handle summary statement scoring, where their behavior diverges from that of baseline models, and which evaluation methods are suitable for comparison.

### Conclusions

We showed that large LLMs can achieve high consistency across repeated runs and moderate to almost perfect agreement with human raters, particularly when few-shot prompting with a CoT initiator is used. The large volume of ratings generated enabled the robust estimation of reliability indices and revealed clear patterns in model- and rubric-level performance.

Our results suggest that LLMs can be used to expand the capacity of VP systems to deliver timely, structured feedback on clinical reasoning for certain rating tasks and for well-defined rubrics. Specifically, they can provide consistent ratings on aspects such as factual accuracy and the inclusion of key patient identifiers, and they show promising, although more variable, performance on rating higher-order reasoning dimensions such as transformation and diagnostic narrowing. This finding indicates that LLM-generated scores could be used to provide immediate, rubric-based feedback after VP cases, helping learners monitor and refine their problem representations more frequently than would be feasible with exclusively human raters. However, their practical use must be further explored. The remaining variability in ternary rubric components, occasional extreme deviations, and rare invalid outputs caution against the use of LLMs as stand-alone graders, especially in summative high-stakes contexts. The opaque nature of LLM decision-making also makes it difficult to justify individual scores to learners. In line with the idea of hybrid intelligence, our findings support an architecture in which LLMs function as supportive tools: they generate rapid and scalable ratings of VP summary statements, whereas human educators can interpret borderline cases, contextualize feedback, and guide learners’ metacognitive reflection on their reasoning. Further refinement of more complex rubric components, such as ternary components that require more reasoning and in-depth understanding (eg, transformation), could help to reduce human involvement in the scoring process.

Future work should examine how students interact with and perceive LLM-generated feedback within VP platforms, whether hybrid human-AI feedback models improve learning outcomes, and how domain-specific adaptations or reasoning models might increase the reliability for abstract reasoning components without compromising transparency. Overall, our findings indicate that with carefully designed prompts and annotated rubrics, current LLMs can support formative feedback on clinical reasoning in VPs, but they remain complementary to expert human judgment.

## Supplementary material

10.2196/94666 Multimedia Appendix 1 Prompt design.

10.2196/94666 Multimedia Appendix 2 The Scoring Rubric.

10.2196/94666 Multimedia Appendix 3 Gwet AC1 and AC2 values.

10.2196/94666 Multimedia Appendix 4 Weighted Cohen Kappa values.

10.2196/94666 Multimedia Appendix 5 Enlarged view of the Claude ratings (top left panel) from Figure 1.

10.2196/94666 Multimedia Appendix 6 Enlarged view of the GPT-4o ratings (top right panel) from Figure 1.

10.2196/94666 Multimedia Appendix 7 Enlarged view of the Mistral Large ratings (bottom left panel) from Figure 1.

10.2196/94666 Multimedia Appendix 8 Enlarged view of the Mistral 7B ratings (bottom right panel) from Figure 1.

10.2196/94666 Multimedia Appendix 9 Distribution of rating scores by the human experts.

10.2196/94666 Multimedia Appendix 10 A collection of 50 exemplary summary statements including the ratings, licensed as CC BY 4.0.

## Abbreviations

AWQ: activation-aware weight quantization
BERT: Bidirectional Encoder Representations from Transformers
CoT: chain-of-thought
HRL: high-resource language
ICC: intraclass correlation coefficient
LLM: large language model
LRM: large reasoning model
NLP: natural language processing
OSCE: Objective Structured Clinical Examination
RQ: research question
SQ: semantic qualifier
VP: virtual patient
