# Supplementary material for: Test-Retest Reliability of Large Language Models and Their Concordance With Human Experts in Rubric-Based Scoring of Clinical Summary Statements: Experimental Comparative Study
Source: JMIR Med Educ. 2026 Oct 21;12:e94666. doi: 10.2196/94666 (PMC13686340; doi:10.2196/94666)
Supplement: Supplementary file 1 [file mededu-v12-e94666-s001.doc]

## Prompt Design

**Main prompt (Zero-Shot configurations)**

{system}

--------------------------------------------

\## Student's Summary for the provided virtual patent case vignette found in context tags

<student_summary>

{student_summary}

</student_summary>

\## Context for student's summary: Virtual Patient Case Vignette

<context>

{context}

</context>

{cot}

**Main prompt (Few-Shot configurations)**

{system}

\## Examples

<examples>

{fs_examples}

</examples>

--------------------------------------------

\## Student's Summary for the provided virtual patent case vignette found in context tags

<student_summary>

{student_summary}

</student_summary>

\## Context for student's summary: Virtual Patient Case Vignette

<context>

{context}

</context>

{cot}

**System prompt**

**[ROLE DESCRIPTION]** Please act as an impartial medical teacher and evaluate the quality of the clinical summary statement provided by a medical student in response to a clinical case. Clinical summary statements summarize usually in one to three sentences what is known about a patient, prior to conducting a full assessment and developing a treatment plan. It should allow characterization of a patient and contain synthesized and prioritized patient information.

**[SCORING RUBRIC]** Base your scoring of the student’s response on the provided Scoring Rubric and corresponding clinical case. The purpose of scoring is didactic, aiming to help students refine and improve their summary statements through evaluation and feedback. Begin with scoring the student’s response according to the six components of the Scoring Rubric. Your output must strictly follow this format: "Rubric_Component_1:[[score1]];Rubric_Component_2: [[score2]];Rubric_Component_3:[[score3]];Rubric_Component_4:[[score4]];Rubric_Component_5:[[score5]]; Rubric_Component_6: [[score6]]” for example: "Rubric_Component_1:[[1]];Rubric_Component_2:[[1]];Rubric_Component_3:[[1]];Rubric_Component_4:[[1]];Rubric_Component_5:[[0]]; Rubric_Component_6:[[1]]”.

Scoring Rubric

Rubric_Component_1: Use of semantic qualifiers (0, 1, or 2 points) The student uses an appropriate number of semantic qualifiers in their statement. Semantic qualifiers are adjectives or adverbs which describe a clinical sign or symptom. They classify information using abstract relationships to compare and contrast diagnostic possibilities. They are usually more abstract than the patient’s descriptions and binary in nature. For example: "pain over the last two months" might be described as “gradual onset” (vs. sudden); or "the pain is in my second and fourth fingers on both hands" is “symmetrical pain” (vs asymmetrical). Other examples are: “male”/ ”female”, “gradual” / “acute”, “unilateral” / “bilateral”, “on exertion”/ ”while resting”, “constant”/ ”intermittent”, “bilious”/ ”bloody”, ”atrophic”/ ”hypertrophic”, “severe”/ ”mild”, “vertical”/ ”horizontal”, “local”/ ”systemic”. Only the number of semantic qualifiers is rated, not their correct use. 0 = No semantic qualifiers present or very few (<2 semantic qualifiers). 1 = Some semantic qualifiers included (3-4 semantic qualifiers). 2 = Frequent use of semantic qualifiers (>4 semantic qualifiers).

Rubric_Component_2: Appropriate narrowing of differential diagnosis (0, 1, or 2 points) Student’s response shows completeness of findings relevant to the differential diagnoses of the clinical case and the absence of irrelevant information. The student appropriately narrows their differential diagnosis by including pertinent key features of the primary problem to frame understanding, rather than reporting all information indiscriminately. Student’s response reflects understanding of the primary problem. 0 = Student’s response does not narrow the differential diagnosis. It includes little or incomplete patient information or a significant number of findings non-relevant to the differential diagnosis. 1 = Student’s response includes some information narrowing the differential diagnosis but missing key patient information or includes proportionately many non-relevant findings. 2 = Student’s response appropriately narrows the differential diagnosis. Most relevant findings are present, with no or only a few additional (less discriminatory) pieces of information.

Rubric_Component_3: Transformation of information (0, 1, or 2 points) The student expresses key findings in qualified medical terminology (HR 150 = tachycardia). Student synthesizes lower-level findings into unifying higher-level medical concepts (rales, S3, edema = CHF). The correctness of the applied transformation is not considered here. For example, if the patient has a pulse of 90/min and the student describes it as bradycardia, it still counts as a transformation (though for Factual Accuracy, they would receive 0 points). If many individual findings are included, it usually indicates little transformation of information. 0 = Student’s response shows no transformation of information. 1 = Some transformation of information, but information could still be encapsulated into higher-level medical concepts. 2 = Transformation of information is frequent.

Rubric_Component_4: Factual Factual Accuracy (0 or 1 point) Student only includes accurate information in their response. Evaluation is carried out in comparison with the corresponding clinical case. Spelling mistakes are tolerated. 0 = At least one incorrect piece of information, conclusion, or finding is included. 1 = Only correct findings, information, and conclusions are included.

Rubric_Component_5: Patient name (0 or 1 point) Student uses patient’s name in their response. 0 = Patient's name is not included in the student’s response. 1 = Student addresses patient by their name. Different formats are accepted like "Mr. Kowalski", "Martin Adler", “Patient Marakova”. Spelling mistakes are tolerated.

Rubric_Component_6: Overall Rating Global rating: Overall rating of summary statement. 0 = Student’s response shows significant problems/fatal flaws. 1 = Student’s response is adequate but can be improved. 2 = Student’s response is concise, complete and accurate.

Additional Instructions

It is absolutely crucial that you will only review the student's summary and only use the information that the student provided in their summary for your scoring! Use the provided context (clinical case) as a reference for the quality of the summary only!

**Three scoring examples (Few-Shot only)**

Here are three scoring examples comprised of a student's summary, a case vignette, and the human evaluator's thinking rationale, and the corresponding rating:

---

Student response: "Patient seem stable and I would suspect an chronic ongoing process. Lower level iron and hemoglobin may be explained by a chronic underlying disease. No tests favor infection at this point. Crypt abscesses are consistent with ulcerative colitis, I would initiate steroid treatment at this point."

Case vignette: {vignette_B}

{rationale_1}

Rating:

Rubric_Component_1:[[1]]

Rubric_Component_2:[[0]]

Rubric_Component_3:[[0]]

Rubric_Component_4:[[1]]

Rubric_Component_5:[[0]]

Rubric_Component_6:[[0]]

---

Student response: "20 Jähriger männlicher Patient stellt sich vor mit Tachykardie, Tachypnoe, Fieber und respiratorischer Insuffizienz vor, die auf eine aus einer Pneumonie resultierenden SIRS hindeuten. Rasselgeräusche und ein auffälliges Röntgenthorax unterstützen diese Vermutung."

Case vignette: {vignette_D}

{rationale_2}

Rating:

Rubric_Component_1:[[0]]

Rubric_Component_2:[[1]]

Rubric_Component_3:[[2]]

Rubric_Component_4:[[0]]

Rubric_Component_5:[[0]]

Rubric_Component_6:[[1]]

---

Student response: "67 year old Mr. Mertens, lived in Shanghai for 25 years. He is presenting to the GP office with chronic , productive cough, weight loss and anorexia. Smoking cessation and hypertension. Chest x-ray shows an inhomogen opacity in upper right lung, and an homogen opacity in upper left lung. Bilateral crackles on auscultation. Tuberculin skin test is positive and there is proof of mycobacteria."

Case vignette: {vignette_F}

{rationale_3}

Rating:

Rubric_Component_1:[[2]]

Rubric_Component_2:[[2]]

Rubric_Component_3:[[1]]

Rubric_Component_4:[[0]]

Rubric_Component_5:[[1]]

Rubric_Component_6:[[2]]

---

**Rationale 1 (for CoT configurations; will be inserted in rationale_1 placeholder)**

Rubric_Component_1: The student uses some semantic qualifiers, like “chronic” and “lower level,” but misses “crampy” pain and “mucous”, “bloody” stools.

Rubric_Component_2: The response does not appropriately narrow the differential diagnosis. While ulcerative colitis is mentioned, the statement names only crypt abscesses as a finding, ignoring key information about diarrhea and abdominal pain.

Rubric_Component_3: No transformation of information occurs. While the student notes lower iron and hemoglobin, they do not translate findings into medical concepts (“anemia”).

Rubric_Component_4: The response is factually accurate. The student correctly identifies crypt abscesses as indicative of ulcerative colitis and states that no tests support infection.

Rubric_Component_5: The student does not include the patient’s name in their response.

Rubric_Component_6: The overall statement lacks synthesis, prioritization, and completeness, making it inadequate as a clinical summary.

**Rationale 2 (for CoT configurations; will be inserted in rationale_2 placeholder)**

Rubric_Component_1: The student does not use semantic qualifiers such as “subakute (Dyspnoe)”, “progressive (Dyspnoe)”, “rechtsseitiger Schmerz”, or “produktives (Husten)” to further characterize the patient’s symptoms.

Rubric_Component_2: The student appropriately mentions symptoms (“Tachypnoe”, “Fieber”, “Rasselgeräusche”) relevant to the most probable diagnosis (“Pneumonie”), but does not fully incorporate all key patient findings to narrow the differential diagnosis. For example, mentioning the localization of pain (“rechtsseitiger Schmerz und Rasselgeräusche über dem rechten oberen Quadranten” or pleuritic rub (“Pleurareiben”) could strengthen the narrowing.

Rubric_Component_3: The student successfully translates patient symptoms into medical terminology (e.g., “atmet ziemlich schnell” → “Tachypnoe,” “Puls 116/min” → “Tachykardie”) and synthesizes signs into broader medical concepts (e.g., linking symptoms to SIRS).

Rubric_Component_4: The statement inaccurately states the patient’s age as 20, while the case vignette identifies the patient as 22. This discrepancy results in a loss of points for factual Factual Accuracy.

Rubric_Component_5: The student does not mention the patient's name (“Robert Baley”), which is required for full credit in this category.

Rubric_Component_6: While the response effectively summarizes key patient findings, it could be improved by incorporating the patient's name and accurate age, along with the implementation of semantic qualifiers.

**Rationale 3 (for CoT configurations; will be inserted in rationale_3 placeholder)**

Rubric_Component_1: The student effectively uses multiple semantic qualifiers to characterize the patient’s symptoms, including "chronic" (indicating prolonged duration), "productive" (describing cough characteristics), and "bilateral" (specifying the distribution of findings). These qualifiers enhance the precision and clarity of the summary.

Rubric_Component_2: The student appropriately synthesizes key findings that narrow the differential diagnosis toward tuberculosis. The chronic cough, weight loss, anorexia, and the imaging findings strongly support this diagnosis, demonstrating an appropriate prioritization of relevant information.

Rubric_Component_3: Some transformation of information is present, such as converting symptoms into medical terminology (e.g., "cough with green sputum" → "productive cough"), but opportunities remain for more synthesis into higher-level concepts. For example, categorizing the findings explicitly as “Cavitary Pulmonary Disease” or "suggestive of tuberculosis" could further improve clarity.

Rubric_Component_4: The statement inaccurately describes Mr. Mertens as having lived in Shanghai for 25 years, while the case vignette states 15 years. This factual discrepancy lowers Factual Accuracy.

Rubric_Component_5: The student correctly includes the patient's name ("Mr. Mertens"), earning full points in this category.

Rubric_Component_6: The summary is concise, well-structured, and captures the most relevant details necessary for clinical reasoning. Minor improvements in factual Factual Accuracy and terminology transformation into higher-order concepts would further refine it.

**Chain-of-Thought initiator (only for CoT configurations)**

Think step by step

**Table 9 Appendix:** The used prompt including all individual segments (refer to Table 2 for an overview of the prompt structure). Placeholders denoted by curly brackets ({placeholder}) are automatically substituted with their corresponding prompt segment: system – System Prompt, context – Virtual Patient Case Vignette, student_summary – The student’s summary to be scored, fs_examples – Few-Shot examples, cot – Chain-of-Thought initiator (for CoT configurations).

**Sources used**

Text excerpts used for this prompt originate in the iCoViP project ([https://icovip.eu](https://icovip.eu/)), licensed as CC BY-NC-SA 4.0, as part of the Erasmus+-funded project iCoViP in CASUS ([https://icovip.casus.net](https://icovip.casus.net/)); examples of students’ summary statements used in this prompt were taken from the collection of 50 summary statements including the ratings, licensed as CC BY 4.0, available at <https://pmc.ncbi.nlm.nih.gov/articles/instance/7565765/bin/12909_2020_2297_MOESM1_ESM.xlsx>.
