# Supplementary material for: Test-Retest Reliability of Large Language Models and Their Concordance With Human Experts in Rubric-Based Scoring of Clinical Summary Statements: Experimental Comparative Study
Source: JMIR Med Educ. 2026 Oct 21;12:e94666. doi: 10.2196/94666 (PMC13686340; doi:10.2196/94666)
Supplement: Supplementary file 2 [file mededu-v12-e94666-s002.doc]

##  The Scoring Rubric

| **Criteria** | **Smith et al.’s (2016) definition [1]** | **Descriptions added by us for the LLMS** | **Scale specifications added by us for LLMs** | **Explanations of and rationales behind the added descriptions** |
| --- | --- | --- | --- | --- |
| **Use of semantic qualifiers** | “Student uses qualitative terms, more abstract than patient’s signs, binary in nature (gradual versus acute, unilateral versus bilateral)  0 = None  1 = Some  2 = Frequent and appropriate” | The student uses an appropriate number of semantic qualifiers in their statement. Semantic qualifiers are adjectives or adverbs which describe a clinical sign or symptom. For example: "pain over the last two months" might be described as “gradual onset” (vs. sudden); or "the pain is in my second and fourth fingers on both hands" is “symmetrical pain” (vs asymmetrical). | 0 = No semantic qualifiers present or very few (<2 semantic qualifiers).  1 = Some semantic qualifiers included (3-4 semantic qualifiers).  2 = Frequent use of semantic qualifiers (>4 semantic qualifiers). | We added examples of semantic qualifiers because LLMs struggled to recognize them. We specified the exact number of SQs according to coding criteria from Hege et al. (2020) [2]. |
| Appropriate narrowing of differential diagnosis | “Student appropriately narrows her/his differential diagnosis by including pertinent key features of the primary problem to frame understanding, rather than reporting all information indiscriminately. Student facilitates understanding of the primary problem.  0 = Does not narrow  1 = Some narrowing but missing elements  2 = Appropriately narrows” | Student’s response shows completeness of findings relevant to the differential diagnoses of the clinical case and the absence of irrelevant information. The student appropriately narrows their differential diagnosis by including pertinent key features of the primary problem | 0 = Student’s response does not narrow the differential diagnosis. It includes little or incomplete patient information or a significant number of findings non-relevant to the differential diagnosis.  1 = Student’s response includes some information narrowing the differential diagnosis but missing key patient information or includes proportionately many non-relevant findings.  2 = Student’s response appropriately narrows the differential diagnosis. Most relevant findings are present, with no or only a few additional (less discriminatory) pieces of information. | LLMs showed a tendency to detect narrowing of the differential diagnosis, where narrowing was not sufficient. Thus, we defined the conditions under which no points should be granted.  The phrase "students response" was included to make it more explicit that the LLM should base its rating on the input student response. (Mistral 7B initially confused context with the summary statement) |
| **Transformation of information** | “Student expresses key findings in qualified medical terminology (HR 150 = tachycardia). Student synthesizes details into unifying medical concepts (rales, S3, edema = CHF)  0 = Non  1 = Some  2 = Frequent and appropriate” | The student expresses key findings in qualified medical terminology. Student synthesizes lower-level findings into unifying higher-level medical concepts. | 0 = Student’s response shows no transformation of information.  1 = Some transformation of information, but information could still be encapsulated into higher-level medical concepts.  2 = Transformation of information is frequent. | This additional description reflects the human rater's initial understanding of this category.  One original rater noted that this criterion is less explicit and more dependent on the internalized knowledge and expertise. |
| **Factual Accuracy** | “Student includes accurate information and does not include misleading information.  0 = No  1 = Yes” | Student only includes accurate information in their response. Evaluation is carried out in comparison with the corresponding clinical case. Spelling mistakes are tolerated. | 0 = At least one incorrect piece of information, conclusion, or finding is included.  1 = Only correct findings, information, and conclusions are included. | Reworded to "at least one incorrect piece of information", because LLMs tended to not grant any points when some clinical information was missing.  Added “in their response” for precision:  LLMs should compare the Factual Accuracy with the context clinical case  “Spelling mistakes are tolerated.”:  LLMs granted zero points when detecting spelling mistakes. According to original raters, Factual Accuracy is not about spelling. |
| **Patient name** | *Not part of Smith et al.’s original rubric but added by Hege et al. (2020)* | Student uses patient’s name in their response. | 0 = Patient's name is not included in the student’s response.  1 = Student addresses patient by their name. Different formats are accepted like "Mr. Kowalski", "Martin Adler", “Patient Marakova”. Spelling mistakes are tolerated again for this binary criterium, LLMs were strictly refraining points when spelling mistakes appeared). | Examples of patient names were added for clarity.  LLMs struggled with German abbreviations; for example, "Pat X" was identified as the students' attempt to provide a name, or assumed that "Pat" was an incorrect name whereas it is just a common abbreviation for “patient”. |
| **Global Rating** | “Overall rating of summary statement.  0 = Siginficant problems/fatal flaws  1 = Adequate but can be improved  2 = Concise, complete, accurate” | Global rating: Overall rating of summary statement. | 0 = Student’s response shows significant problems/fatal flaws.  1 = Student’s response is adequate but can be improved.  2 = Student’s response is concise, complete and accurate. | This category remained unchanged. In the view of one of the original human raters it is a holistic, largely subjective scoring category which was hard to define more precisely and explicitely. |

**Table 8 Appendix:** The rubric as used in the experiments. The table compares Hege et al.’s (2020) adopted version of Smith et al.’s (2016) summary statement evaluation rubric, the instructions and explanations we iteratively developed to optimise its applicability to LLMs, and a short rationale for each.

## References

1. Smith S, Kogan JR, Berman NB, Dell MS, Brock DM, Robins LS. The Development and Preliminary Validation of a Rubric to Assess Medical Students’ Written Summary Statements in Virtual Patient Cases: *Academic Medicine*. 2016;91(1):94-100. doi:[10.1097/ACM.0000000000000800](https://doi.org/10.1097/ACM.0000000000000800)
2. Hege I, Kiesewetter I, Adler M. Automatic analysis of summary statements in virtual patients - a pilot study evaluating a machine learning approach. *BMC Med Educ*. 2020;20(1):366. doi:[10.1186/s12909-020-02297-w](https://doi.org/10.1186/s12909-020-02297-w)
