# Supplementary material for: Test-Retest Reliability of Large Language Models and Their Concordance With Human Experts in Rubric-Based Scoring of Clinical Summary Statements: Experimental Comparative Study
Source: JMIR Med Educ. 2026 Oct 21;12:e94666. doi: 10.2196/94666 (PMC13686340; doi:10.2196/94666)
Supplement: Supplementary file 3 [file mededu-v12-e94666-s003.doc]

## Gwet’s AC1/AC2

|  | | | **Gwet’s AC1/AC2** | | | |
| --- | --- | --- | --- | --- | --- | --- |
| **Model** | Configuration | Rubric Component | Mean | SE | Lower | Upper |
| **Claude** | Few-Shot | Factual Accuracy | 0,578 | 0,004 | 0,571 | 0,586 |
|  | Few-Shot | Global | 0,802 | 0,002 | 0,799 | 0,805 |
|  | Few-Shot | Narrowing | 0,634 | 0,002 | 0,631 | 0,637 |
|  | Few-Shot | Person | 0,961 | 0,004 | 0,954 | 0,968 |
|  | Few-Shot | SQ | 0,475 | 0,000 | 0,475 | 0,475 |
|  | Few-Shot | Transformation | 0,769 | 0,002 | 0,765 | 0,774 |
|  | Few-Shot CoT | Factual Accuracy | 0,707 | 0,000 | 0,707 | 0,707 |
|  | Few-Shot CoT | Global | 0,818 | 0,000 | 0,818 | 0,818 |
|  | Few-Shot CoT | Narrowing | 0,701 | 0,000 | 0,701 | 0,701 |
|  | Few-Shot CoT | Person | 0,970 | 0,000 | 0,970 | 0,970 |
|  | Few-Shot CoT | SQ | 0,705 | 0,002 | 0,702 | 0,708 |
|  | Few-Shot CoT | Transformation | 0,740 | 0,001 | 0,738 | 0,743 |
|  | Zero-Shot | Factual Accuracy | 0,683 | 0,000 | 0,683 | 0,683 |
|  | Zero-Shot | Global | 0,810 | 0,000 | 0,810 | 0,810 |
|  | Zero-Shot | Narrowing | 0,559 | 0,000 | 0,559 | 0,559 |
|  | Zero-Shot | Person | 0,985 | 0,000 | 0,985 | 0,985 |
|  | Zero-Shot | SQ | 0,334 | 0,000 | 0,334 | 0,334 |
|  | Zero-Shot | Transformation | 0,409 | 0,000 | 0,409 | 0,409 |
|  | Zero-Shot CoT | Factual Accuracy | 0,649 | 0,003 | 0,643 | 0,654 |
|  | Zero-Shot CoT | Global | 0,801 | 0,000 | 0,801 | 0,801 |
|  | Zero-Shot CoT | Narrowing | 0,577 | 0,000 | 0,577 | 0,577 |
|  | Zero-Shot CoT | Person | 0,985 | 0,000 | 0,985 | 0,985 |
|  | Zero-Shot CoT | SQ | 0,372 | 0,000 | 0,372 | 0,372 |
|  | Zero-Shot CoT | Transformation | 0,466 | 0,000 | 0,466 | 0,466 |
| **GPT-4o** | Few-Shot | Factual Accuracy | 0,800 | 0,004 | 0,792 | 0,807 |
|  | Few-Shot | Global | 0,729 | 0,005 | 0,719 | 0,739 |
|  | Few-Shot | Narrowing | 0,685 | 0,003 | 0,679 | 0,690 |
|  | Few-Shot | Person | 0,898 | 0,011 | 0,877 | 0,919 |
|  | Few-Shot | SQ | 0,384 | 0,006 | 0,373 | 0,396 |
|  | Few-Shot | Transformation | 0,528 | 0,002 | 0,524 | 0,533 |
|  | Few-Shot CoT | Factual Accuracy | 0,880 | 0,007 | 0,866 | 0,894 |
|  | Few-Shot CoT | Global | 0,749 | 0,004 | 0,742 | 0,756 |
|  | Few-Shot CoT | Narrowing | 0,678 | 0,005 | 0,669 | 0,687 |
|  | Few-Shot CoT | Person | 0,858 | 0,004 | 0,850 | 0,865 |
|  | Few-Shot CoT | SQ | 0,518 | 0,004 | 0,511 | 0,525 |
|  | Few-Shot CoT | Transformation | 0,652 | 0,012 | 0,627 | 0,676 |
|  | Zero-Shot | Factual Accuracy | 0,817 | 0,006 | 0,806 | 0,829 |
|  | Zero-Shot | Global | 0,641 | 0,005 | 0,631 | 0,651 |
|  | Zero-Shot | Narrowing | 0,640 | 0,004 | 0,632 | 0,649 |
|  | Zero-Shot | Person | 0,899 | 0,003 | 0,894 | 0,904 |
|  | Zero-Shot | SQ | 0,257 | 0,004 | 0,248 | 0,266 |
|  | Zero-Shot | Transformation | 0,235 | 0,006 | 0,222 | 0,247 |
|  | Zero-Shot CoT | Factual Accuracy | 0,828 | 0,009 | 0,811 | 0,846 |
|  | Zero-Shot CoT | Global | 0,638 | 0,003 | 0,633 | 0,644 |
|  | Zero-Shot CoT | Narrowing | 0,638 | 0,001 | 0,636 | 0,640 |
|  | Zero-Shot CoT | Person | 0,894 | 0,005 | 0,884 | 0,905 |
|  | Zero-Shot CoT | SQ | 0,277 | 0,004 | 0,268 | 0,286 |
|  | Zero-Shot CoT | Transformation | 0,224 | 0,005 | 0,214 | 0,234 |
| **Mistral Large** | Few-Shot | Factual Accuracy | 0,530 | 0,008 | 0,514 | 0,546 |
|  | Few-Shot | Global | 0,852 | 0,007 | 0,839 | 0,866 |
|  | Few-Shot | Narrowing | 0,767 | 0,010 | 0,747 | 0,787 |
|  | Few-Shot | Person | 0,693 | 0,010 | 0,674 | 0,712 |
|  | Few-Shot | SQ | 0,749 | 0,011 | 0,728 | 0,770 |
|  | Few-Shot | Transformation | 0,786 | 0,006 | 0,774 | 0,798 |
|  | Few-Shot CoT | Factual Accuracy | 0,848 | 0,007 | 0,833 | 0,862 |
|  | Few-Shot CoT | Global | 0,890 | 0,004 | 0,882 | 0,897 |
|  | Few-Shot CoT | Narrowing | 0,842 | 0,007 | 0,829 | 0,855 |
|  | Few-Shot CoT | Person | 0,932 | 0,007 | 0,917 | 0,946 |
|  | Few-Shot CoT | SQ | 0,768 | 0,011 | 0,746 | 0,790 |
|  | Few-Shot CoT | Transformation | 0,807 | 0,003 | 0,800 | 0,813 |
|  | Zero-Shot | Factual Accuracy | 0,691 | 0,013 | 0,667 | 0,716 |
|  | Zero-Shot | Global | 0,914 | 0,003 | 0,908 | 0,919 |
|  | Zero-Shot | Narrowing | 0,792 | 0,005 | 0,783 | 0,801 |
|  | Zero-Shot | Person | 0,970 | 0,000 | 0,970 | 0,970 |
|  | Zero-Shot | SQ | 0,820 | 0,004 | 0,811 | 0,828 |
|  | Zero-Shot | Transformation | 0,744 | 0,005 | 0,733 | 0,754 |
|  | Zero-Shot CoT | Factual Accuracy | 0,692 | 0,010 | 0,673 | 0,711 |
|  | Zero-Shot CoT | Global | 0,921 | 0,002 | 0,917 | 0,925 |
|  | Zero-Shot CoT | Narrowing | 0,796 | 0,006 | 0,784 | 0,809 |
|  | Zero-Shot CoT | Person | 0,976 | 0,004 | 0,969 | 0,983 |
|  | Zero-Shot CoT | SQ | 0,817 | 0,006 | 0,806 | 0,829 |
|  | Zero-Shot CoT | Transformation | 0,746 | 0,006 | 0,735 | 0,757 |
| **Mistral 7B Local** | Few-Shot | Factual Accuracy | 0,449 | 0,011 | 0,427 | 0,472 |
|  | Few-Shot | Global | 0,694 | 0,016 | 0,663 | 0,726 |
|  | Few-Shot | Narrowing | 0,465 | 0,010 | 0,446 | 0,485 |
|  | Few-Shot | Person | 0,292 | 0,039 | 0,215 | 0,370 |
|  | Few-Shot | SQ | 0,753 | 0,028 | 0,698 | 0,807 |
|  | Few-Shot | Transformation | 0,763 | 0,012 | 0,740 | 0,787 |
|  | Few-Shot CoT | Factual Accuracy | 0,721 | 0,014 | 0,693 | 0,748 |
|  | Few-Shot CoT | Global | 0,693 | 0,015 | 0,664 | 0,722 |
|  | Few-Shot CoT | Narrowing | 0,675 | 0,010 | 0,655 | 0,694 |
|  | Few-Shot CoT | Person | 0,173 | 0,013 | 0,147 | 0,199 |
|  | Few-Shot CoT | SQ | 0,810 | 0,007 | 0,797 | 0,823 |
|  | Few-Shot CoT | Transformation | 0,794 | 0,016 | 0,762 | 0,826 |
|  | Zero-Shot | Factual Accuracy | 0,840 | 0,000 | 0,840 | 0,840 |
|  | Zero-Shot | Global | 0,386 | 0,016 | 0,355 | 0,416 |
|  | Zero-Shot | Narrowing | 0,365 | 0,016 | 0,334 | 0,396 |
|  | Zero-Shot | Person | 0,037 | 0,027 | -0,016 | 0,089 |
|  | Zero-Shot | SQ | 0,876 | 0,026 | 0,825 | 0,927 |
|  | Zero-Shot | Transformation | 0,601 | 0,020 | 0,562 | 0,639 |
|  | Zero-Shot CoT | Factual Accuracy | 0,840 | 0,000 | 0,840 | 0,840 |
|  | Zero-Shot CoT | Global | 0,516 | 0,015 | 0,487 | 0,546 |
|  | Zero-Shot CoT | Narrowing | 0,411 | 0,023 | 0,366 | 0,456 |
|  | Zero-Shot CoT | Person | 0,103 | 0,025 | 0,053 | 0,152 |
|  | Zero-Shot CoT | SQ | 0,832 | 0,035 | 0,765 | 0,900 |
|  | Zero-Shot CoT | Transformation | 0,550 | 0,028 | 0,495 | 0,606 |

**Table 5 Appendix:** *Gwet’s AC1/AC2* inter-rater agreement coefficients for the four LLMs across four prompting configurations (Zero-Shot, Few-Shot, and with / without Chain-of-Thought) and the six rubric components, showing mean agreement scores with 95% confidence intervals (n=5 repeated runs for each combination).

|  |  | Gwet’s AC1/AC2 | | | | |
| --- | --- | --- | --- | --- | --- | --- |
| Model | Configuration | Mean | SD | SE | Lower | Upper |
| Claude | Few-shot | 0.70 | 0.16 | 0.03 | 0.64 | 0.76 |
|  | Few-shot CoT | 0.77 | 0.1 | 0.02 | 0.74 | 0.81 |
|  | Zero-shot | 0.63 | 0.23 | 0.04 | 0.54 | 0.72 |
|  | Zero-shot CoT | 0.64 | 0.21 | 0.04 | 0.56 | 0.72 |
| GPT-4o | Few-shot | 0.67 | 0.17 | 0.03 | 0.61 | 0.74 |
|  | Few-shot CoT | 0.72 | 0.13 | 0.02 | 0.68 | 0.77 |
|  | Zero-shot | 0.58 | 0.26 | 0.05 | 0.48 | 0.68 |
|  | Zero-shot CoT | 0.58 | 0.26 | 0.05 | 0.49 | 0.68 |
| Mistral Large | Few-shot | 0.73 | 0.10 | 0.02 | 0.69 | 0.77 |
|  | Few-shot CoT | 0.85 | 0.06 | 0.01 | 0.83 | 0.87 |
|  | Zero-shot | 0.82 | 0.10 | 0.02 | 0.79 | 0.86 |
|  | Zero-shot CoT | 0.82 | 0.10 | 0.02 | 0.79 | 0.86 |
| Mistral 7B local | Few-shot | 0.57 | 0.19 | 0.03 | 0.5 | 0.64 |
|  | Few-shot CoT | 0.64 | 0.22 | 0.04 | 0.56 | 0.73 |
|  | Zero-shot | 0.52 | 0.30 | 0.05 | 0.41 | 0.63 |
|  | Zero-shot CoT | 0.54 | 0.26 | 0.05 | 0.44 | 0.64 |

**Table 6 Appendix:** *Gwet’s AC1/AC2* inter-rater agreement coefficients for the four LLMs across four prompting configurations (Zero-Shot, Few-Shot, and with / without Chain-of-Thought), showing mean agreement scores over the six rubric components with 95% confidence intervals (n=5 repeated runs for each combination).
