# Supplementary material for: Test-Retest Reliability of Large Language Models and Their Concordance With Human Experts in Rubric-Based Scoring of Clinical Summary Statements: Experimental Comparative Study
Source: JMIR Med Educ. 2026 Oct 21;12:e94666. doi: 10.2196/94666 (PMC13686340; doi:10.2196/94666)
Supplement: Supplementary file 4 [file mededu-v12-e94666-s004.doc]

## Weighted Cohen’s κ

|  | | | **Weighted Cohen’s κ** | | | |
| --- | --- | --- | --- | --- | --- | --- |
| **Model** | Configuration | Rubric Component | Mean | SE | Lower | Upper |
| **Claude** | Few-Shot | Factual Accuracy | 0,253 | 0,003 | 0,248 | 0,258 |
|  | Few-Shot | Global | 0,680 | 0,001 | 0,678 | 0,682 |
|  | Few-Shot | Narrowing | 0,586 | 0,001 | 0,584 | 0,588 |
|  | Few-Shot | Person | 0,953 | 0,004 | 0,944 | 0,962 |
|  | Few-Shot | SQ | 0,457 | 0,000 | 0,457 | 0,457 |
|  | Few-Shot | Transformation | 0,609 | 0,003 | 0,604 | 0,613 |
|  | Few-Shot CoT | Factual Accuracy | 0,328 | 0,000 | 0,328 | 0,328 |
|  | Few-Shot CoT | Global | 0,645 | 0,000 | 0,645 | 0,645 |
|  | Few-Shot CoT | Narrowing | 0,634 | 0,000 | 0,634 | 0,634 |
|  | Few-Shot CoT | Person | 0,964 | 0,000 | 0,964 | 0,964 |
|  | Few-Shot CoT | SQ | 0,557 | 0,002 | 0,554 | 0,560 |
|  | Few-Shot CoT | Transformation | 0,615 | 0,002 | 0,611 | 0,618 |
|  | Zero-Shot | Factual Accuracy | 0,274 | 0,000 | 0,274 | 0,274 |
|  | Zero-Shot | Global | 0,551 | 0,000 | 0,551 | 0,551 |
|  | Zero-Shot | Narrowing | 0,481 | 0,000 | 0,481 | 0,481 |
|  | Zero-Shot | Person | 0,982 | 0,000 | 0,982 | 0,982 |
|  | Zero-Shot | SQ | 0,380 | 0,000 | 0,380 | 0,380 |
|  | Zero-Shot | Transformation | 0,392 | 0,000 | 0,392 | 0,392 |
|  | Zero-Shot CoT | Factual Accuracy | 0,211 | 0,002 | 0,207 | 0,216 |
|  | Zero-Shot CoT | Global | 0,546 | 0,000 | 0,546 | 0,546 |
|  | Zero-Shot CoT | Narrowing | 0,505 | 0,000 | 0,505 | 0,505 |
|  | Zero-Shot CoT | Person | 0,982 | 0,000 | 0,982 | 0,982 |
|  | Zero-Shot CoT | SQ | 0,393 | 0,000 | 0,393 | 0,393 |
|  | Zero-Shot CoT | Transformation | 0,426 | 0,000 | 0,426 | 0,426 |
| **GPT-4o** | Few-Shot | Factual Accuracy | 0,299 | 0,006 | 0,288 | 0,310 |
|  | Few-Shot | Global | 0,589 | 0,005 | 0,580 | 0,598 |
|  | Few-Shot | Narrowing | 0,581 | 0,003 | 0,576 | 0,586 |
|  | Few-Shot | Person | 0,877 | 0,014 | 0,850 | 0,903 |
|  | Few-Shot | SQ | 0,386 | 0,004 | 0,378 | 0,394 |
|  | Few-Shot | Transformation | 0,468 | 0,003 | 0,463 | 0,473 |
|  | Few-Shot CoT | Factual Accuracy | 0,502 | 0,022 | 0,458 | 0,545 |
|  | Few-Shot CoT | Global | 0,609 | 0,004 | 0,601 | 0,617 |
|  | Few-Shot CoT | Narrowing | 0,591 | 0,004 | 0,583 | 0,600 |
|  | Few-Shot CoT | Person | 0,832 | 0,004 | 0,823 | 0,841 |
|  | Few-Shot CoT | SQ | 0,438 | 0,003 | 0,433 | 0,444 |
|  | Few-Shot CoT | Transformation | 0,528 | 0,010 | 0,508 | 0,548 |
|  | Zero-Shot | Factual Accuracy | 0,361 | 0,005 | 0,352 | 0,371 |
|  | Zero-Shot | Global | 0,569 | 0,004 | 0,561 | 0,576 |
|  | Zero-Shot | Narrowing | 0,571 | 0,004 | 0,562 | 0,579 |
|  | Zero-Shot | Person | 0,874 | 0,003 | 0,868 | 0,881 |
|  | Zero-Shot | SQ | 0,323 | 0,004 | 0,315 | 0,330 |
|  | Zero-Shot | Transformation | 0,349 | 0,002 | 0,344 | 0,353 |
|  | Zero-Shot CoT | Factual Accuracy | 0,415 | 0,026 | 0,363 | 0,466 |
|  | Zero-Shot CoT | Global | 0,575 | 0,001 | 0,572 | 0,577 |
|  | Zero-Shot CoT | Narrowing | 0,579 | 0,002 | 0,575 | 0,583 |
|  | Zero-Shot CoT | Person | 0,867 | 0,007 | 0,853 | 0,881 |
|  | Zero-Shot CoT | SQ | 0,333 | 0,003 | 0,327 | 0,339 |
|  | Zero-Shot CoT | Transformation | 0,344 | 0,002 | 0,340 | 0,347 |
| **Mistral Large** | Few-Shot | Factual Accuracy | 0,106 | 0,013 | 0,080 | 0,132 |
|  | Few-Shot | Global | 0,627 | 0,008 | 0,612 | 0,641 |
|  | Few-Shot | Narrowing | 0,613 | 0,013 | 0,588 | 0,638 |
|  | Few-Shot | Person | 0,558 | 0,015 | 0,528 | 0,588 |
|  | Few-Shot | SQ | 0,473 | 0,012 | 0,448 | 0,497 |
|  | Few-Shot | Transformation | 0,520 | 0,007 | 0,506 | 0,533 |
|  | Few-Shot CoT | Factual Accuracy | 0,222 | 0,021 | 0,182 | 0,262 |
|  | Few-Shot CoT | Global | 0,657 | 0,007 | 0,642 | 0,672 |
|  | Few-Shot CoT | Narrowing | 0,637 | 0,013 | 0,612 | 0,663 |
|  | Few-Shot CoT | Person | 0,916 | 0,009 | 0,898 | 0,935 |
|  | Few-Shot CoT | SQ | 0,504 | 0,027 | 0,450 | 0,557 |
|  | Few-Shot CoT | Transformation | 0,568 | 0,005 | 0,558 | 0,579 |
|  | Zero-Shot | Factual Accuracy | 0,124 | 0,018 | 0,089 | 0,160 |
|  | Zero-Shot | Global | 0,698 | 0,008 | 0,682 | 0,714 |
|  | Zero-Shot | Narrowing | 0,655 | 0,004 | 0,647 | 0,662 |
|  | Zero-Shot | Person | 0,964 | 0,000 | 0,964 | 0,964 |
|  | Zero-Shot | SQ | 0,520 | 0,010 | 0,500 | 0,540 |
|  | Zero-Shot | Transformation | 0,471 | 0,006 | 0,459 | 0,483 |
|  | Zero-Shot CoT | Factual Accuracy | 0,124 | 0,014 | 0,096 | 0,151 |
|  | Zero-Shot CoT | Global | 0,716 | 0,004 | 0,709 | 0,723 |
|  | Zero-Shot CoT | Narrowing | 0,653 | 0,010 | 0,635 | 0,672 |
|  | Zero-Shot CoT | Person | 0,971 | 0,004 | 0,963 | 0,980 |
|  | Zero-Shot CoT | SQ | 0,509 | 0,012 | 0,484 | 0,533 |
|  | Zero-Shot CoT | Transformation | 0,482 | 0,010 | 0,462 | 0,501 |
| **Mistral 7B Local** | Few-Shot | Factual Accuracy | 0,067 | 0,017 | 0,033 | 0,101 |
|  | Few-Shot | Global | 0,198 | 0,011 | 0,175 | 0,220 |
|  | Few-Shot | Narrowing | 0,184 | 0,014 | 0,156 | 0,212 |
|  | Few-Shot | Person | 0,260 | 0,034 | 0,193 | 0,327 |
|  | Few-Shot | SQ | 0,225 | 0,025 | 0,176 | 0,274 |
|  | Few-Shot | Transformation | 0,373 | 0,032 | 0,310 | 0,435 |
|  | Few-Shot CoT | Factual Accuracy | 0,256 | 0,030 | 0,196 | 0,315 |
|  | Few-Shot CoT | Global | 0,399 | 0,015 | 0,369 | 0,429 |
|  | Few-Shot CoT | Narrowing | 0,453 | 0,022 | 0,409 | 0,497 |
|  | Few-Shot CoT | Person | 0,259 | 0,011 | 0,238 | 0,280 |
|  | Few-Shot CoT | SQ | 0,274 | 0,022 | 0,231 | 0,317 |
|  | Few-Shot CoT | Transformation | 0,456 | 0,044 | 0,369 | 0,542 |
|  | Zero-Shot | Factual Accuracy | 0,000 | 0,000 | 0,000 | 0,000 |
|  | Zero-Shot | Global | 0,172 | 0,015 | 0,144 | 0,201 |
|  | Zero-Shot | Narrowing | 0,206 | 0,015 | 0,177 | 0,235 |
|  | Zero-Shot | Person | 0,156 | 0,022 | 0,112 | 0,200 |
|  | Zero-Shot | SQ | 0,169 | 0,017 | 0,135 | 0,204 |
|  | Zero-Shot | Transformation | 0,192 | 0,026 | 0,140 | 0,244 |
|  | Zero-Shot CoT | Factual Accuracy | 0,000 | 0,000 | 0,000 | 0,000 |
|  | Zero-Shot CoT | Global | 0,251 | 0,013 | 0,225 | 0,276 |
|  | Zero-Shot CoT | Narrowing | 0,255 | 0,025 | 0,207 | 0,304 |
|  | Zero-Shot CoT | Person | 0,207 | 0,021 | 0,166 | 0,248 |
|  | Zero-Shot CoT | SQ | 0,194 | 0,030 | 0,135 | 0,252 |
|  | Zero-Shot CoT | Transformation | 0,206 | 0,036 | 0,136 | 0,276 |

**Table 7 Appendix:** Weighted *Cohen’s κ* for the four LLMs across four prompting configurations (Zero-Shot, Few-Shot, and with / without Chain-of-Thought) and the six rubric components, showing mean agreement scores with 95% confidence intervals (n=5 repeated runs for each combination).
