# Supplementary material for: Test-Retest Reliability of Large Language Models and Their Concordance With Human Experts in Rubric-Based Scoring of Clinical Summary Statements: Experimental Comparative Study
Source: JMIR Med Educ. 2026 Oct 21;12:e94666. doi: 10.2196/94666 (PMC13686340; doi:10.2196/94666)
Supplement: Supplementary file 5 [file mededu-v12-e94666-s009.doc]

**Figure 5 Appendix:** Distribution of rating scores by the human experts for 125 summary statements written by medical students as taken from the dataset by Hege and colleagues [15]. The chart shows the proportion of 0,1 and 2 score ratings over the six rubric components.
